# Hormone-mediated neural remodeling orchestrates parenting onset during pregnancy

**DOI:** 10.1126/science.adi0576

**Published:** 2023-10-05

**Authors:** Rachida Ammari, Francesco Monaca, Mingran Cao, Estelle Nassar, Patty Wai, Nicholas A. Del Grosso, Matthew Lee, Neven Borak, Deborah Schneider-Luftman, Johannes Kohl

**Affiliations:** 1 State-dependent Neural Processing Laboratory, The Francis Crick Institute, 1 Midland Rd, London NW1 1AT, UK; 2 Bioinformatics and Biostatistics Science Technology Platform, The Francis Crick Institute, 1 Midland Rd, London NW1 1AT, UK

## Abstract

During pregnancy physiological adaptations prepare the female body for the challenges of motherhood. Becoming a mother also requires behavioral adaptations. Such adaptations can already occur during pregnancy, but how pregnancy hormones remodel parenting circuits to instruct preparatory behavioral changes remains unknown. We found that action of estradiol and progesterone on galanin (Gal)-expressing neurons in the mouse medial preoptic area (MPOA) is critical for pregnancy-induced parental behavior. While estradiol silences MPOA^Gal^ neurons and paradoxically increases their excitability, progesterone permanently rewires this circuit node by promoting dendritic spine formation and recruitment of excitatory synaptic inputs. This MPOA^Gal^-specific neural remodeling sparsens population activity *in vivo* and results in persistently stronger, more selective responses to pup stimuli. Pregnancy hormones thus remodel parenting circuits in anticipation of future behavioral need.

Motherhood is associated with pronounced behavioral changes in many species, such as altered feeding routines and increased aggressivity (1–9). These adaptations are typically attributed to the hormonal changes associated with giving birth (parturition), which have been hypothesized to activate or ‘prime’ parental circuits (10,11). One of the most striking differences between sexually inexperienced (virgin) females and mothers is their infant-directed behaviors: while virgins typically avoid infants or exhibit low levels of parental behavior, mothers are highly parental (12–14). Classical studies in rats found increased maternal responsiveness during pregnancy (9, 15, 16). This even occurs in females undergoing a caesarean section during mid- or late pregnancy, and persists for weeks (15, 17–20). Correspondingly, parental behavior can be elicited in virgin rats by mimicking the hormonal changes of pregnancy (21–27), which include drastic rises in the levels of estradiol (E2) and progesterone (P4) (fig. S1A). These observations indicate hormone-mediated, preparatory neural adaptations to infant-directed behavior during pregnancy. However, despite the identification of numerous forms of pregnancy-associated neural plasticity (1, 28, 29), it remains unknown how pregnancy hormones affect parenting circuits to mediate changes in infant-directed behavior.

## Hormone-dependent, long-lasting changes to pup-directed behavior in pregnancy

**Fig. 1:**
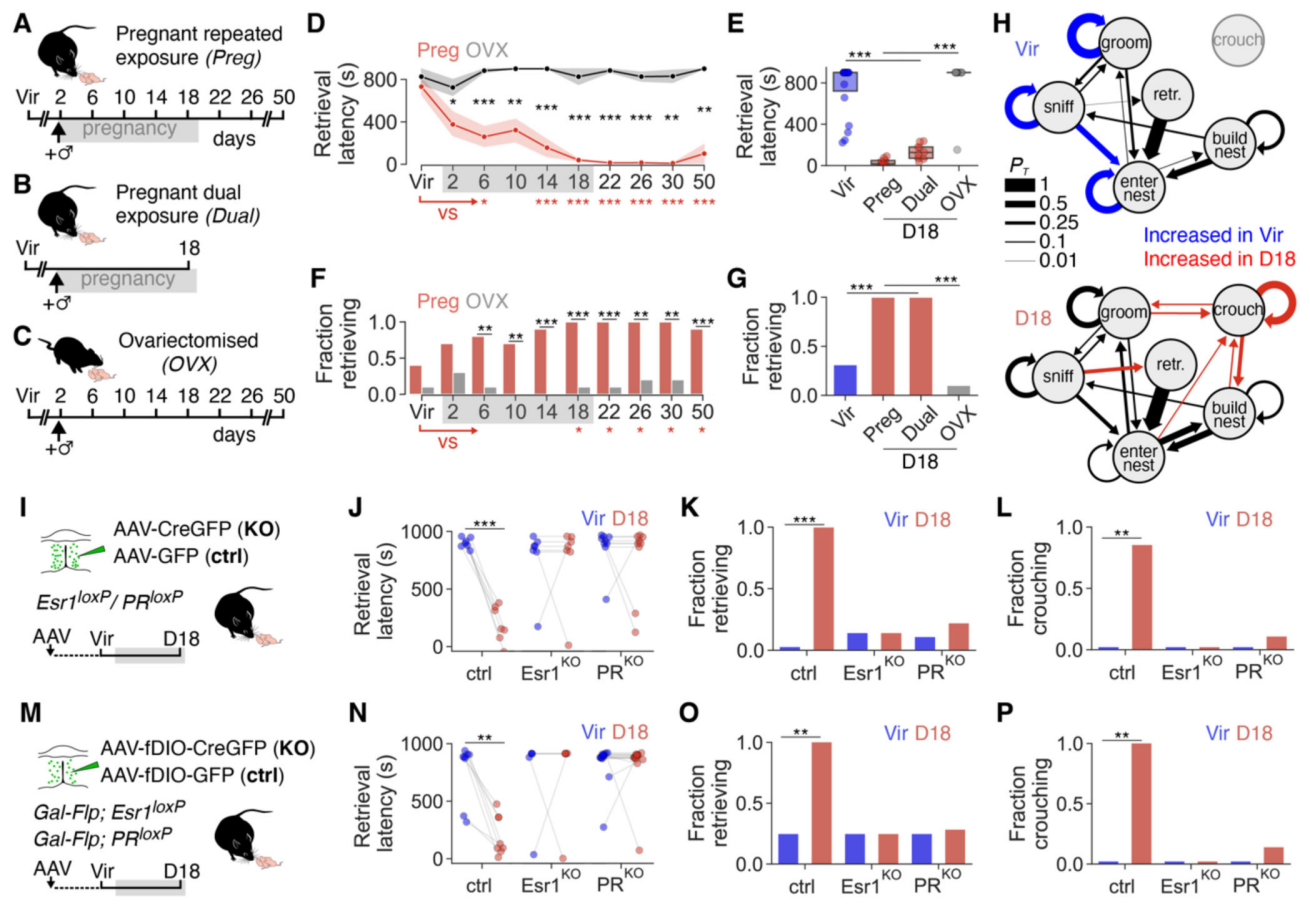
Hormone action on MPOA^Gal^ neurons is critical for pregnancy-induced onset of parental behavior. (**A** to **C**) Testing pup-directed behavior in repeatedly pup-exposed pregnant females (**A,** *Preg*, n = 10), pregnant females exposed to pups twice (**B**, *Dual*, n = 9) and repeatedly pup-exposed ovariectomized females (**C,** *OVX*, n = 10). Day of pregnancy (A and B) or relative to pairing with male (C) shown. (**D** and **F**) Parental behaviors in *Preg* group. Within-group *(Preg*, virgin [Vir] vs each subsequent timepoint, red asterisks) and between-group *(Preg* vs *OVX*, black asterisks) shown. (**E** and **G**) Comparison of Vir and D18 timepoints across groups. Note that virgins from *Preg* and *Dual* groups are pooled (fig. S1M). (**H**) Behavioral state transition diagrams for Vir and D18 females (*Preg*, n = 10). Average transition probabilities (*P_T_*) between behaviors are shown, and differences between Vir and D18 highlighted if *P* < 0.05 (U test, see materials and methods). (**I**) AAV-mediated ablation (KO) of Esr1 or PR in MPOA, and control (ctrl). (**J** to **L**) Effects of MPOA-wide KO of Esr1 or PR on pup-directed behaviors (n = 7, 8, 9 mice). (**M**) KO of Esr1 or PR in MPOA^Gal^ neurons. (**N** to **P**) Effects of MPOA^Gal^- specific KO of Esr1 or PR on pup-directed behaviors (n = 8, 5, 13 mice). Kaplan-Meier survival analysis with log rank test in (D), (E), (J) and (N), Fisher’s exact test with Benjamini-Hochberg adjustment for multiple comparisons in (F), (G), (K), (L), (O) and (P). Shaded area in (D) is SEM. ***P < 0.001, **P < 0.01, *P < 0.05.

Whereas virgin female rats and wild house mice typically ignore or attack pups, respectively, virgin female laboratory mice often exhibit hormone-independent, spontaneous parental behavior (9). We first asked when and how pup interactions change during pregnancy. We exposed female mice to pups at regular intervals before, during and after pregnancy, and scored their behaviors *(Preg*, Fig. 1A). Most aspects of parental behavior were affected by pregnancy (Fig. 1, D to G and fig. S1, B to J) and this was particularly pronounced in late pregnant females (day 18, D18): all D18 females retrieved pups with short latency (D18: 39.7 ± 10.8 s, virgins: 477.9 ± 143.3 s, Fig. 1, D to G), crouched above pups (17.3 ± 3.5% of assay duration) and spent most of their time in the nest (fig. S1, B and C). In addition to individual aspects of parenting, pregnancy affected behavioral sequences: whereas D18 females performed sequences of retrieval, crouching, nest building and grooming, virgins engaged in repetitive sniffinggrooming-nest entering episodes (Fig. 1H). The increased parental performance of D18 females could be due to hormonal effects and/or frequent pup exposure (30). We therefore assessed pup interactions in females that were exposed to pups only as virgins and at D18 *(Dual*, Fig. 1B) and in ovariectomized females *(OVX*, Fig. 1C) (31). Pup retrieval, crouching and time in nest differed between virgins (Vir) and D18 in the *Preg* and *Dual* groups. In contrast, such differences were not present in the *OVX*group over similar time points, and are thus primarily affected by pregnancy hormones (Fig. 1, D to G and fig. S1, B to E). These behaviors were also upregulated in females that were pup-exposed only once, at D18, illustrating that the pregnancy-induced onset of parenting does not require any previous pup exposure (fig. S1, N to S). The hormonal milieu of pregnancy thus leads to an onset of specific parental behaviors in mice, and these behavioral changes are maximal in late pregnancy. Most behavioral changes persisted until at least one month after parturition (D50, Fig. 1, D and F and fig. S1D), when hormone levels have returned to baseline (fig. S1A). These adaptations thus likely result from long-lasting remodeling of the brain by pregnancy hormones.

## Hormone action on MPOA^Gal^ neurons is critical for parenting onset

Parenting is controlled by brain-wide circuits (32–36), several elements of which might be affected by hormones. In particular, the MPOA - which is critical for parental behavior - has been shown to be a hormonal target (37–39). Parental behavior can be induced in virgin female rats by administration of the hormones E2, P4, prolactin and oxytocin (21–26, 40, 41), and global knockout of their canonical receptors impairs parenting (42–45). Because combined systemic administration of E2 and P4 is most effective in triggering parenting onset (12), the underlying neural substrates are likely sensitive to both hormones. E2 and P4 can permanently modulate neuronal function via intracellular receptors that act as transcription factors (46–49). We focused on the intracellular estrogen receptor 1 (Esr1) and progesterone receptor (PR), because they are critical for parental behavior (43, 50–52) and because the long-lasting nature of pregnancy-induced behavioral changes implicates gene expression-dependent forms of plasticity. Using single-molecule fluorescent *in situ* hybridization (smFISH) from hypothalamic brain sections of virgins and D18 females, we found that Esr1/PR co-expressing neurons (as well as neurons expressing either receptor) were most enriched in MPOA subregions (fig. S2, A to F). Prolactin receptor, but not oxytocin receptor, expression was similarly enriched in the MPOA (fig. S2, G and H).

To determine whether Esr1 or PR expression in the MPOA is required for parenting onset during pregnancy, mice carrying floxed receptor alleles (see materials and methods and ref. (53)) were injected into the MPOA with an adeno-associated virus (AAV) expressing Cre recombinase (Fig. 1I). This resulted in local receptor knockout (KO), whereas injection of a GFP-expressing control AAV did not affect receptor expression (fig. S3, A to H). MPOA-specific ablation of either Esr1 or PR had no effect on pup interactions in virgins but completely blocked the pregnancy-induced upregulation of pup retrieval, crouching and nest time at D18 (Fig. 1, J to L and fig. S4, A to C). In contrast, parental behaviors were normally upregulated at D18 in animals injected with control AAVs (Fig. 1, J to L and fig. S4, A to D).

Several overlapping populations of MPOA neurons are involved in the control of parenting, with galanin- expressing (MPOA^Gal^) neurons being critical for this behavior (33–35, 54–56). MPOA^Gal^ neurons, most of which express Esr1 and PR (fig. S3, J and K), constitute ~20% of MPOA neurons (35). To determine whether hormonal sensitivity of this subpopulation is necessary for pregnancy-induced behavioral adaptations, we made a knock-in mouse line expressing Flp recombinase in galanin neurons (fig. S3I and materials and methods) and crossed this allele into mice with floxed receptor genes. AAV-mediated ablation of either Esr1 or PR in MPOA^Gal^ neurons fully recapitulated the effects observed after MPOA- wide receptor KO (Fig. 1, M to P and fig. S4, E to H). In contrast, pup contact latency, a parameter not modulated by pregnancy, was not affected by this manipulation (fig. S4I). The parental behaviors of these receptor-ablated animals remained impaired even after giving birth (D22, fig. S5), indicating that the lack of hormone-mediated behavioral preparations during pregnancy cannot be compensated for by the subsequent endocrine events of parturition. Direct action of E2 and P4 on MPOA^Gal^ neurons via their intracellular hormone receptors is therefore necessary for pregnancy-mediated increases in parental behavior.

## Long-lasting hormonal remodeling of MPOA^Gal^ neurons during pregnancy

**Fig. 2:**
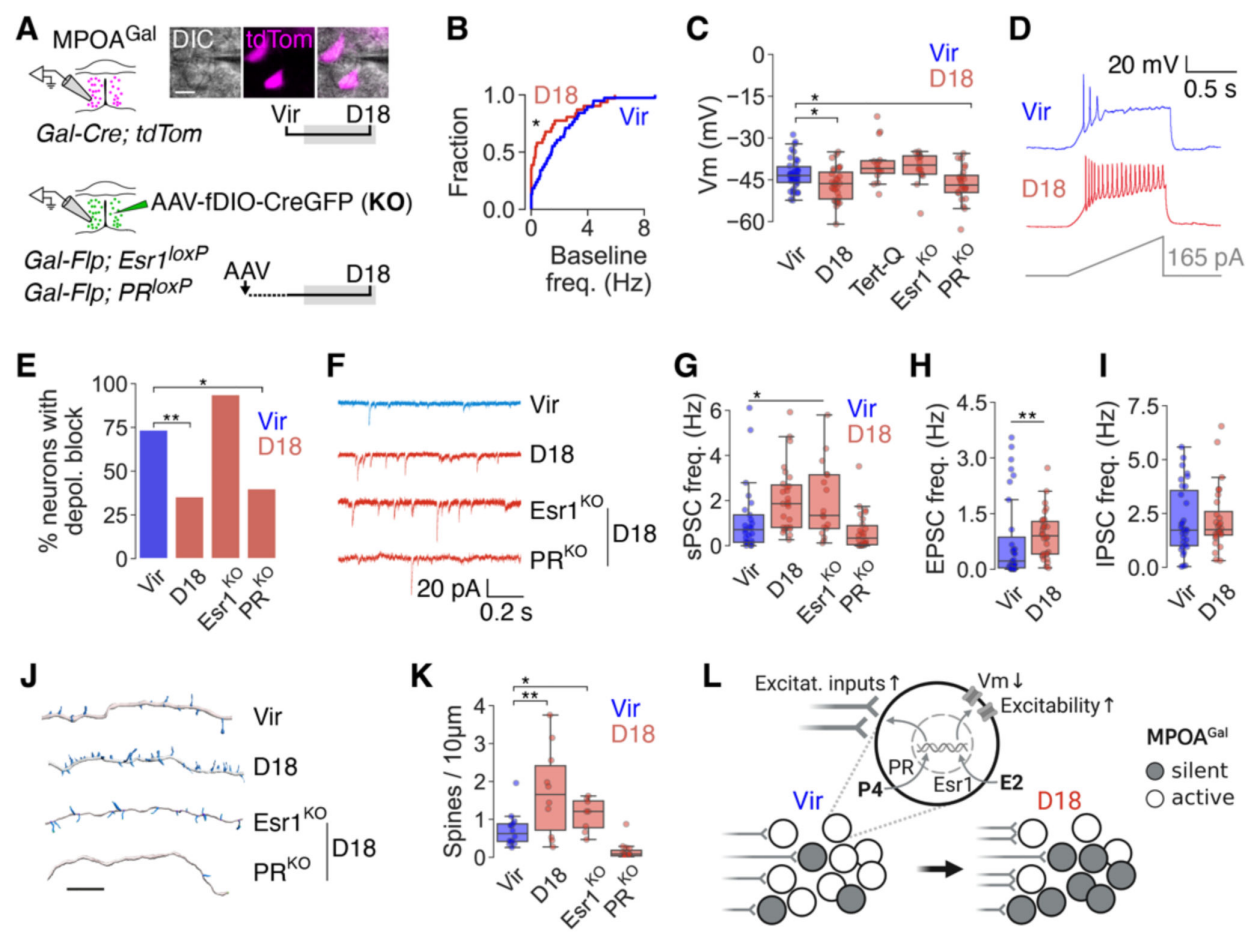
Hormonal remodeling of MPOA^Gal^ neurons. (**A**) Whole-cell recordings from wild-type *(upper panel)* and receptor-deleted *(bottompanel*, KO) MPOA^Gal^ neurons. (**B**) Cumulative distribution of baseline firing frequency (Vir, D18; 33, 21 cells from n = 15, 7 mice). (**C**) Resting membrane potential of control and receptor-deleted MPOA^Gal^ neurons, and recordings in presence of GIRK channel blocker Tertiapin-Q (Tert-Q) (38, 32, 15, 18, 26 cells from n = 15, 9, 3, 3, 5 mice). (**D**) Example current clamp recording traces of cells with (Vir) and without (D18) depolarization block. (**E**) Percentage of neurons exhibiting depolarization block (34, 30, 18, 25 cells from n = 15, 8, 3, 5 mice). (**F**) Example voltage clamp recording traces with sPSCs. (**G**) sPSC frequency (21, 23, 18, 26 cells from n = 9, 6, 3, 5 mice). (**H** and **I**) EPSC (H, Vir, D18; 31, 30 cells from n = 5, 4 mice) and IPSC (I, 31, 28 cells from n = 5, 4 mice) frequency. (**J**) Dendritic segments of MPOA^Gal^ neurons with spines. (**K**) Spine density (14, 10, 8, 15 cells from n = 10, 4, 3, 4 mice). (**L**) Summary scheme for hormonal remodeling of MPOA^Gal^ neurons. U test in (B). One-way ANOVA with Dunnett’s *post hoc* test in (C), (G) and (K). Fisher’s exact test with Benjamini-Hochberg adjustment in (E). K-S test in (H) and (I). Scale bars, 20 μm (A), 10 μm (J). ***P < 0.001, **P < 0.01, *P < 0.05.

We next asked how pregnancy affects MPOA^Gal^ neurons and performed patch clamp recordings in brain slices from virgins and D18 females (Fig. 2A, *upper panel).* Recorded neurons were Neurobiotin-filled and reconstructed to assess morphological changes. MPOA^Gal^ neurons exhibited lower baseline firing and resting membrane potential in late pregnancy (Fig. 2, B and C), with a significantly higher proportion of silent neurons at D18 (fig. S6, A and B). This silencing was abolished by Tertiapin-Q (TQ) and might thus be mediated by GIRK channels (Fig. 2C). At the same time, MPOA^Gal^ neurons were more excitable at D18, and less frequently exhibited depolarization block (Fig. 2, D and E and fig. S6K). We observed a reduction in action potential half-width at D18 (fig. S6E), hinting at increased function of delayed rectifier K^+^ channels which repolarize neurons to permit sustained firing (57). These effects on neuronal membrane properties were already apparent in mid-pregnancy (D10, fig. S6, B to E) and were linked, because 80% of silent MPOA^Gal^ neurons also did not exhibit depolarization block at D18 (fig. S6O). MPOA^Gal^ silencing was not due to increased inhibitory synaptic inputs: although these neurons received more spontaneous postsynaptic currents (sPSCs) at D18, this was due to an increase in excitatory inputs (Fig. 2, F to I and fig. S6M) which predominantly targeted spontaneously active, i.e. non-silenced, neurons (fig. S6N). Correspondingly, MPOA^Gal^ neurons had more dendritic spines at D18 (Fig. 2, J and K). This remodeling of synaptic inputs was also already detectable a D10 (fig. S6, I and J). We did not observe changes to sPSC amplitude and dynamics (fig. S6L), or to dendritic complexity and somatic volume (fig. S6, P and Q) (58). Pregnancy therefore reduces the baseline activity of MPOA^Gal^ neurons, while increasing their excitability and promoting the recruitment of excitatory synaptic inputs. Pregnancy did not have equivalent effects on Gal-negative MPOA neurons, highlighting the specificity of MPOA^Gal^ neuronal remodeling (fig. S7).

To address whether these biophysical and morphological changes were due to direct hormonal action, we recorded from MPOA^Gal^ neurons in which Esr1 or PR were deleted (Fig. 2A, *lower panel).* Ablation of these receptors returned specific, non-overlapping aspects of D18 neuronal physiology to a virgin-like state: Esr1 deletion specifically prevented pregnancy-induced silencing and changes to excitability (Fig. 2, C and E) but did not affect synaptic inputs and spine density (Fig. 2, F, G, J and K). In contrast, PR deletion selectively abolished the upregulation of synaptic inputs and spine density (Fig. 2, G and K), without affecting membrane properties (Fig. 2, C and E). Transduction with a control AAV had no effect (fig. S6, R to W). E2 and P4 therefore control discrete aspects of pregnancy-induced plasticity in MPOA^Gal^ neurons: while E2 tunes membrane potential and intrinsic excitability, P4 mediates the recruitment of additional excitatory synaptic inputs (Fig. 2L). To assess how long-lasting these changes were, we recorded from MPOA^Gal^ neurons in mothers shortly after parturition (D22) and at D50, when pregnancy- and parturition-associated hormone levels have returned to baseline (fig. S8A). MPOA^Gal^ resting membrane potential and firing frequency remained reduced at D22 and only returned to virgin-like levels at D50 (fig. S8, B and C), whereas neuronal excitability reverted immediately after parturition (fig. S8, E and F). In contrast, synaptic inputs and spine density showed a long-lasting upregulation (fig. S8, G and H). Similar to the lasting behavioral effects of receptor ablation, its physiological effects persisted in mothers (fig. S8, I to K). These observations suggest that pregnancy hormones permanently alter the circuit integration of MPOA^Gal^ neurons, thereby providing a cellular substrate for the long-lasting behavioral effects of pregnancy.

## Reorganization of MPOA^Gal^ neuronal and neural population activity during pregnancy

**Fig. 3:**
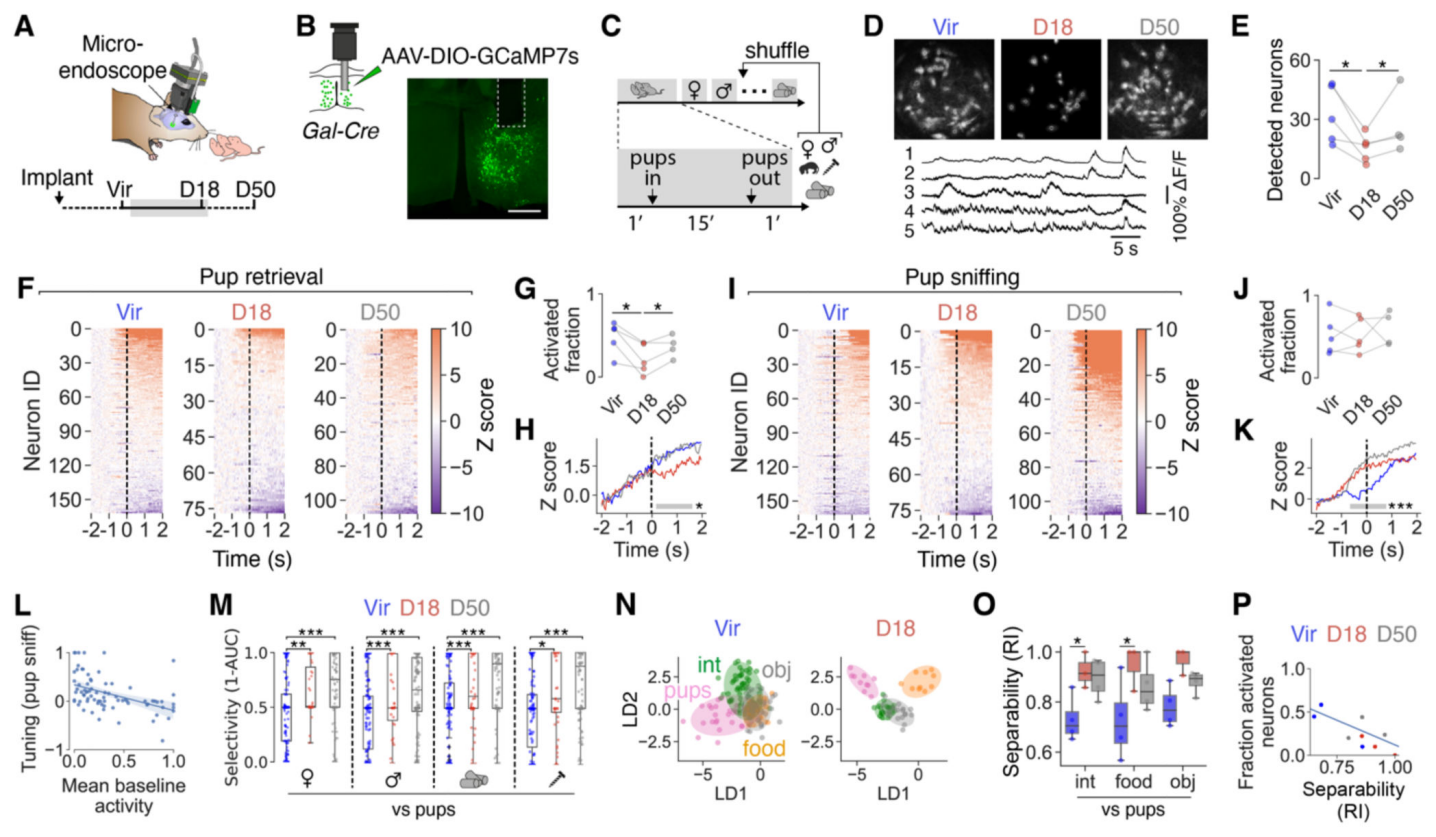
Reorganization of MPOA^Gal^ population activity during pregnancy. (**A**) Recording setup for miniature microscope recordings. (**B**) *Gal-Cre* animals were injected into the MPOA with AAV-FLEx- GCaMP7s and implanted with a GRIN lens. GCaMP7s expression and GRIN lens position shown. (C) Experimental design (see materials and methods). (**D**) Sample recording frames with detected neurons and example activity traces from a virgin. (**E**) Number of detected (non-silent) neurons per animal (n = 5 mice). (**F** and **I**) Temporal profile of MPOA^Gal^ responses during pup retrieval (F) or sniffing (I) in virgins, at D18 and D50 (162, 77, 93 neurons from n = 5 mice). Dashed lines indicate action onset. Order based on hierarchical clustering sorted by mean cluster response onset. (**G** and **J**) Fraction of neurons with positive evoked response during pup retrieval (G) or sniffing (J, n = 5 mice). (**H** and **K**) Averaged Z score for neurons activated during pup retrieval in virgins, at D18 and D50 (H, 115, 41, 63 neurons from 5, 5, 4 mice) or sniffing (K, 122, 51, 86 neurons from 5, 5, 4 mice). Two-way ANOVA with Tukey *post hoc* test; gray bars indicate periods of significant difference for Vir vs D18 and Vir vs D50. (**L**) Correlation between normalized tuning index for responses to pup sniffing and normalized mean baseline activities at D18 (r^2^ = 0.202, *P* < 2.4 × 10^-5^). (**M**) Selectivity of chemoinvestigation-associated responses for indicated stimulus pairs at Vir, D18 and D50 (142, 35, 108 cells from n = 4, 3, 4 mice) compared to pups. A selectivity score of 1 means the neuron is only activated during pup sniffing, a score of 0 means selective activation during sniffing of other stimulus, and 0.5 equals a non-selective response (see materials and methods). (**N**) Example MPOA^Gal^ neuronal activity at Vir and D18 during object investigation in LDA space (int, intruder; obj, screw, dummy pup). Temporal bins were used as features. Ellipsoids represent 95% confidence area of neuronal activity to each stimulus. (**O**) Separability of indicated stimulus combinations by the MPOA^Gal^ population (RI, Rand Index, n = 4, 3, 4 mice). (**P**) Correlation between separability and activated fraction of neurons during pup retrieval (r^2^ = 0.56, *P* < 6.1 × 10^-21^). Paired *t* tests in (E), (G) and (J), mixed linear model with mouse ID as group in (M). Linear regression in (L) and (P), unpaired *t* tests in (O). Scale bar in (B) 500 μm. ***P < 0.001, **P < 0.01, *P < 0.05.

We next investigated the effects of pregnancy on MPOA^Gal^ neural activity *in vivo.* We performed longitudinal, cellular-resolution calcium imaging from MPOA^Gal^ neurons in females exposed to pups and a set of social and non-social stimuli (Fig. 3, A to C and fig. S9A) (59). Consistent with the silencing observed in our slice physiology recordings, the number of detectable (non-silent) MPOA^Gal^ neurons was significantly reduced at D18 *in vivo* (Fig. 3, D and E). This reduction was not due to a decline in the number of GCaMP-expressing MPOA^Gal^ neurons over time, or increased calcium buffering by rising GCaMP levels, because it was reversible (Fig. 3E) and did not occur in virgin females recorded at identical time points (fig. S9B). It also did not result from potential shifts in the recording plane, because we observed this effect when imaging *ex vivo* (fig. S9, E and F). Finally, the number of detected neurons was not significantly decreased in MPOA-wide recordings (fig. S9, C and D). Pregnancy-induced silencing therefore preferentially occurs in MPOA^Gal^ neurons, consistent with our electrophysiological findings in brain slices (fig. S7, A to E).

The fraction of neurons activated during pup retrieval and pup grooming decreased at D18 (Fig. 3, F and G and fig. S10A). In contrast, similar fractions of MPOA^Gal^ neurons were active during pup sniffing in virgins and at D18, but their responses occurred with shorter latency at D18 and D50 (Fig. 3, I to K), indicating a higher excitability of MPOA^Gal^ neurons to pup stimuli during and after pregnancy. Pregnancy therefore sparsens MPOA^Gal^ population activity during parental actions and makes these neurons more excitable to pup stimuli. The baseline activity of individual MPOA^Gal^ neurons was negatively correlated with their tuning to pup stimuli at D18, thereby linking neuronal silencing to stronger pup-evoked responses (Fig. 3L). To address pregnancy-induced differences in how MPOA^Gal^ neurons represent pup stimuli, we examined their activity patterns during chemoinvestigation of pups and other stimuli (Fig. 3C). MPOA^Gal^ neuronal stimulus selectivity for, and response strength to, pups increased in late pregnancy (Fig. 3M and fig. S11, A to E). Similarly, while linear discriminant analysis (LDA) could not separate pup representations in MPOA^Gal^ neurons well from those of other stimuli in virgins, separability of pup representations from those of other stimuli was enhanced at D18 (Fig. 3, N and O). Stimulus separability was positively correlated with population sparsening, thereby linking improved pup representations with effective encoding of parental actions (Fig. 3P). At D50, the numbers of spontaneously active and retrieval-activated neurons had largely returned to virgin levels (Fig. 3, E and G), mirroring our findings in brain slices (fig. S8, B to D). In contrast, pup stimulus selectivity and separability showed a long-lasting increase (Fig. 3, M to O). These findings demonstrate that pregnancy leads to a pronounced sparsening of spontaneous and parenting-associated activity in MPOA^Gal^ neurons, and to increased selectivity for infant stimuli.

## Discussion

Considerable progress has been made in uncovering the functional architecture of parenting circuits (9, 32–36), but little is known about how hormones alter these circuits to ensure state-dependent behavioral flexibility. We discovered that pregnancy hormone action on MPOA^Gal^ neurons — a hub in parenting circuits — is critical to instruct a preparatory change in infant-directed behavior. The ovarian hormones E2 and P4 each control distinct aspects of pregnancy-induced neural remodeling: while E2 transiently silences MPOA^Gal^ neurons and increases their excitability, P4 permanently remodels this circuit element by recruiting synaptic inputs. This results in sparsened population activity during parental behavior, and in potentiated, more selective responses to pup stimuli. We propose that the resulting increase in signal-to-noise, both in individual neurons and at population level, enables more efficient encoding of parental motor actions by MPOA^Gal^ neurons. Population sparsening via silencing might contribute to setting up the circuit for efficient parental behavior by selectively recruiting inputs onto active MPOA^Gal^ neurons during pregnancy. Once rewired, this circuit could then drive robust parenting in response to pup cues, whereas release from silencing during the postpartum period might allow for recruitment of these neurons during non-parental social interactions.

The long-lasting, P4-mediated remodeling of MPOA^Gal^ synaptic inputs provides a cellular correlate for the long-lasting behavioral changes we observe. Although parturition-associated hormonal changes and subsequent maternal experience cannot compensate for lack of hormonal remodeling during pregnancy, these factors might normally contribute to long-term enhancement of maternal behavior (‘maternal memory’) (9). Repeated and/or prolonged co-housing of virgins with pups results in elevated levels of parenting through sensitization (60, 61), and parental care can be socially transmitted by mothers (62). It is unclear whether these paradigms result in similar neuronal changes. Ablating MPOA^Gal^ neurons or making them hormone-insensitive both abolish pup retrieval, but optogenetic activation of these neurons elicits pup grooming in virgins (35). While it remains unknown which neuronal ensembles are recruited by artificial, acute stimulation, they seem to differ from the sparse populations that drive robust parenting in late pregnancy.

E2 silences MPOA^Gal^ neurons beyond parturition, presumably by upregulating GIRK channel expression, whereas the more transient increases in excitability are likely due to potentiated function of delayed rectifier K^+^ channels (57). The identity of the additional excitatory inputs recruited by P4 remains unknown. They might constitute long-range afferents conveying pup sensory information because the majority of local MPOA neurons are GABAergic (54). Future work will characterize the identity and functional role of the cellular pathways targeted by Esr1 and PR. Co-expression of these receptors is not unique to Gal-expressing MPOA neurons (fig. S2C). We hypothesize that permissive chromatin states in these neurons allow for cell-type specific hormonally induced target gene expression. MPOA^Gal^ neurons form molecularly distinct subpopulations (54) which might be differentially affected by pregnancy hormones. We also expect MPOA^Gal^ and other neurons in parenting circuits to be sensitive to additional pregnancy hormones such as prolactin, placental lactogens, allopregnanolone and oxytocin. Prolactin for instance, which acts on the MPOA in early pregnancy to reduce physical activity (63) might also contribute to early changes in pup-directed behavior (Fig. 1, D and F).

Unlike laboratory mice, the majority of wild virgin female mice exhibit infanticide (64). Our work provides mechanisms through which hormones might act in parental circuits of wild mice and other species that critically depend on endocrine changes for the onset of short-latency maternal behavior, such as rats, rabbits and sheep (9). The neural activity changes observed here — i.e. population sparsening and increased stimulus selectivity and discriminability — are reminiscent of changes occurring during critical periods in the developing brain (65). Our work therefore suggests that pregnancy hormones open a window of adult plasticity during which neural remodeling orchestrates behavioral adaptations for the future challenges of motherhood.

## Supplementary Material

Movie S1

Supplementary Material

## Data Availability

The data that support the findings of this study will be made publicly available on Figshare (10.25418/crick.c.6706164). Larger files (e.g. videos) will be made available by the corresponding author upon reasonable request. Original data are stored on the Crick file server. All code will be made available on GitHub: (https://github.com/FrancisCrickInstitute/AmmariMonaca2023).

## One-Sentence Summary

Hormonal remodeling of distinct hypothalamic neurons orchestrates the onset of parental behavior already during pregnancy.
