# Supplementary material for: Hormone-mediated neural remodeling orchestrates parenting onset during pregnancy
Source: Science. Author manuscript; Available in PMC 2023 Nov 1. (PMC7615220; doi:10.1126/science.adi0576)
Supplement: Supplementary Material [file EMS189416-supplement-Supplementary_Material.pdf]

Science AAAS

Supplementary Materials for

Hormone-mediated neural remodeling orchestrates parenting onset during pregnancy

Rachida Ammari[1,†], Francesco Monaca[1,†], Mingran Cao[1], Estelle Nassar[1], Patty Wai[1], Nicholas A. Del Grosso[1], Matthew Lee[1], Neven Borak[1], Deborah Schneider-Luftman[2] & Johannes Kohl[1*]

Corresponding author: jonny.kohl@crick.ac.uk

The PDF file includes:

Materials and Methods
Supplementary Text
Figs. S1 to S11
References

Other Supplementary Materials for this manuscript include the following:

Movie S1

Materials and Methods

Ethical compliance

All animal procedures performed in this study were approved by the UK government (Home Office) and by the Crick Institutional Animal Welfare Ethical Review Panel.

Mice

Animals were housed in individually ventilated cages on a 12 h:12 h light : dark cycle (light on: 22:00–10:00) with food and water available ad libitum. All pregnant mice in this study were primiparous. Timed pregnancies were set up during mid-dark phase (16:00) and females were checked for vaginal plugs every morning on subsequent days. Gestation length amounts to 19.3 days in the C57BL/6J strain (*66*). Pregnancy Day (D) 0 was defined by when a vaginal plug was detected. Postpartum time points (e.g. D22, D50) are thus indicated from pregnancy onset. For pup-directed behavior experiments, C57BL/6J mice from the Crick breeding colonies were used at age 8–14 weeks. The Gal-Cre BAC transgenic line (Stock: Tg(Gal-cre) KI87Gsat/Mmucd, 031060-UCD) was imported from the Mutant Mouse Regional Resource Center. For slice physiology and slice calcium imaging experiments, this line was crossed to Cre-dependent Rosa26 Tomato (Ai9, JAX #007909) or RCL-GCaMP6f (Ai95, JAX #028865) reporter mice, respectively. The Gal-Flp line was generated by CRISPR/Cas9-mediated knock-in of a P2A-FlpO cassette 3' of the STOP codon (Exon 6) of the galanin gene (Biocytogen). For hormone receptor KO experiments, this line was crossed to Esr1^{loxP} (estrogen receptor α conditional knockout, imported from EMMA, EM:11179) (*53*) or PR^{loxP} (progesterone receptor conditional knockout). The PR^{loxP} line was generated by Crick Genetic Modification Services (GeMS) using CRISPR/Cas9-mediated gene targeting. Two pairs of single guide RNAs (sgRNAs, IDT Alt-R CRISPR-Cas9 sgRNA, 2 nmol) and single-stranded oligo DNA nucleotides (ssODNs, IDT Ultramer DNA Oligo, 4 nmol) containing two loxP sites were designed to target intronic regions flanking exon 2 of the *Pgr* gene. Zygotes obtained from superovulation of C57BL/6J mice were electroporated with 50 µl of a mix of Cas9 protein (1.2 µM), sgRNA (6.0 µM) and ssODN (8.0 µM) and transferred into pseudopregnant mice. Mutant F0 mice were identified by genotyping (Transnetyx qPCR) and NGS sequencing of PCR amplicons (Illumina MiSeq 250bp paired end reads). After a further cross to C57BL/6J, F1 mice were mated to a PGK-Cre line (JAX

#000664) and Cre-mediated excision confirmed in the offspring. All mouse lines used in this study were backcrossed to C57BL/6J.

<u>Behavioral profiling</u>

Experimental groups

In the pregnant repeated exposure *(Preg)* group, virgin females were tested 7 days before being paired up with an experienced stud male. Pup interactions (see 'Pup-directed behavior assay') were assessed every 4 days throughout pregnancy. After giving birth, females were co-housed with their litters since pup removal can result in dysfunctional social behavior (*67*), but litters were removed before the habituation period of each behavioral test. Pups were weaned at 21 days. In the ovariectomized *(OVX)* group, animals were allowed to recover for 7 days before behavioral testing. OVX females were then paired up with stud males, and behavioral testing was otherwise identical. In the dual exposure group *(Dual)*, females underwent behavioral testing only twice, in the virgin state one week before mating, and at D18. In the single exposure group (*1x*), females were only tested at D18.

Pup-directed behavior assay

Animals were individually housed 7 days before behavioral testing. Experiments were performed in the first half of the dark phase in the animal's home cage and were preceded by a 10 min habituation period. Two 1–3 day old C57BL/6 J foster pups (randomly chosen from litters of 5–8) were placed in different corners opposite the nest, and pup interactions were video recorded for 15 min with a camera (Basler Ace GigE, acA1300-60gmNIR) capturing a top view of the cage at a frame rate of 30 Hz. Manual scoring was performed using EthoVision XT 13 (Noldus). Manually segmented behaviors were analyzed using custom Python scripts. Duration of behaviors is reported as percentage of total assay duration. Latency of behaviors was calculated from introduction of the first pup into the cage. Pup-directed behaviors were quantified as follows: Contact latency was defined as the first contact of the animal's nose with a pup. Pup grooming was defined as close contact with pups, involving licking, pup displacement and rhythmic head movements. Pup chemoinvestigation was defined as close interaction without contact. Retrieval latency was defined by the first pup being picked up and successfully carried to the nest. Fraction of mice retrieving or crouching refers to the fraction of mice that retrieve, or crouch over, at least one pup. Time

in nest was defined as the time the female stayed in the nest with at least one pup. Crouching was defined as the female stationarily positioned over pup(s) in the nest. Nest building was defined as gathering bedding/nesting material to an existing nest or shaping it into a new nest. Nest scores were assessed at the start of each assay during the habituation phase and defined as follows: Score 0 – no nest, scattered nesting material; 1 – flat nest; 2 – plate-shaped nest with low walls; 3 – plate-shaped nest with higher walls; 4 – fully closed, dome-shaped nest (see (*8*)). Behavior transition probabilities were calculated as follows: temporally ordered lists of scored behaviors were concatenated for virgins (n = 10) and pregnant females (D18, n = 10) from the *Preg* group. Both lists were filtered for relevant behaviors, and the behaviors 'digging' and 'self-grooming' were classified as 'other'. Behaviors were then split into pairs *(behavior 1, behavior 2)* and the number of occurrences for a given pair divided by the total number of pairs with the same *behavior 1* to obtain the probability for this given pair of behaviors to occur. Note that not all possible behavior transitions are shown in the diagrams. U tests were performed for each behavior pair between Vir and D18, and differences highlighted if $P < 0.05$.

<u>Surgical procedures and viral injections</u>

Analgesia was provided on the day before surgery (0.15 ml carprofen in 200 ml drinking water). Mice were anaesthetized using isoflurane (4% for induction, 1.5% for maintenance) in oxygen-enriched air and then head-fixed on a stereotaxic frame (Model 940, Kopf Instruments). Meloxicam (10 mg / kg body weight) and Buprenorphine (0.1 mg / kg body weight) were given subcutaneously before craniotomy. Surgery sites were closed using Vicryl sutures (Ethicon) or Vetbond (3M). Carprofen was provided in drinking water for 2 days following surgery for postoperative pain management. Eyes were protected with ophthalmic ointment (Viscotears, Alcon). Rectal body temperature was maintained at 37 °C during surgery using a heating pad (Harvard Apparatus) and animals were kept in a heated recovery chamber until mobile.

Deep brain imaging

AAV2/1-syn-FLEx-jGCaMP7s-WPRE (Addgene 104491), or AAV2/1-syn-GCaMP7s (custom construct, packaged by Crick Vector Core) was unilaterally injected into the MPOA of Gal-Cre or C57BL/6J mice, respectively, using a Nanoject II or Nanoject III injector (Drummond Scientific) and pulled glass

capillaries (3-000-203-G/X, Drummond Scientific). AAVs were injected at volumes of 100–200 nl at a titer of 2×10^{13} GC / ml. MPOA injection coordinates were AP 0.0 mm (from bregma), ML ± 0.5 mm (from midline), DV −5.05 mm (from pia). After letting the virus diffuse for 5 min, the injection needle was slowly retracted and an integrated gradient-index lens (0.6 × 7.3 mm, 1050-002177, Inscopix) was slowly implanted at a depth of −4.95 mm (from brain surface) and fixed to the skull using UV light-curable glue (RelyX Unicem, 3M) and Superbond cement (Prestige Dental). Recordings started 6–8 weeks after surgery.

Receptor knockout experiments

For MPOA-wide receptor KO experiments, $Esr1^{loxP}$ or PR^{loxP} mice received bilateral injections of either AAV2/5-CMV-EGFP-Cre (250 nl, Addgene 105545, 2×10^{13} GC / ml) or AAV2/5-CMV-EGFP (250 nl, Addgene 105530, 2×10^{13} GC / ml). For $MPOA^{Gal}$-specific manipulations and recordings, the following viruses were used: AAV2/1-syn-fDIO-EGFP-2A-iCre (250 nl, custom construct, packaged by Vectorbuilder, 4×10^{13} GC / ml) or AAV2/1-syn-fDIO-EGFP (250nl, custom construct, packaged by Vectorbuilder, 2×10^{13} GC / ml) injected into the MPOA (coordinates as above) of Gal-Flp, $Esr1^{loxP}$ or Gal-Flp, PR^{loxP} mice. Animals were tested two weeks after injection as virgins, and at D18. Receptor KO efficiency was assessed in a separate cohort of animals that received unilateral MPOA injections (see ‘Imaging and image analysis’).

<u>Histology and immunostaining</u>

Animals were perfused transcardially with phosphate-buffered saline (PBS) followed by 4% paraformaldehyde (PFA) in PBS. Brains were dissected and post-fixed in 4% PFA o/n, then washed in PBS. After embedding in 4% low-melting point agarose (Thermo Fisher, 16520-050) in PBS, 60 µm coronal sections were cut on a vibratome (Leica) and mounted on Superfrost Plus slides (VWR, 48311-703) with DAPI-containing Vectashield mounting medium (Vector Laboratories, H-1200). For immunostaining in 48-well culture plates, sections were permeabilized for 30 min in PBS-T (0.3% Triton X-100 in PBS), post-fixed with 4% PFA in PBS for 10 min, and washed in PBS-T (3 × 20 min). Blocking was carried out for 2 h at room temperature in blocking buffer (0.3% Triton X-100, 1% BSA, 2% normal donkey serum in PBS). Incubation with primary antibody (rabbit anti-Esr1, Millipore 06-935, 1:5,000)

was performed for 24 h on a nutator at 4°C. After washing in PBS-T (5 × 60 min), secondary antibody (Alexa Fluor-488 anti-rabbit, Thermo Fisher A-11008, 1:1,500) was added for 48 h at 4°C. After washes in PBS-T (5 × 60 min), NeuroTrace 530/615 Red Fluorescent Nissl Stain (Thermo Fisher N21482, 1:100) was added for 30 min at RT. Sections were washed in PBS-T for a final round (3 × 20 min) and mounted.

<u>Hormone receptor mapping</u>

In situ hybridization and immunostaining

Animals were perfused transcardially with ice-cold PBS, and freshly dissected brains embedded in OCT (Tissue-Tek, 4583), frozen on dry ice and stored at −80°C. Subsequently, 18 μm cryosections were cut on a Leica CM1950 cryostat and collected on Superfrost Plus slides (VWR, 48311-703) in three series, only one of which was stained and imaged. Slides were fixed in 10% Neutral buffered formalin (NBF), followed by a series of dehydration steps in ethanol (5 min each of 50%, 70%, 100%, 100% v/v EtOH). Slides were pre-treated with RNAscope Protease III reagent. Single-molecule fluorescent *in situ* hybridization (smFISH) and immunohistochemistry (IHC) against NeuN were performed on slides using the RNAscope LS Multiplex Reagent Kit (Advanced Cell Diagnostics), LS 4-Plex Ancillary Kit and Multiplex Reagent Kit on a robotic staining system (Leica BOND-III). RNAscope probes were Esr1 (ACD, Cat. No. 475248), Pgr-C2 (Cat. No. 318928-C2, detects PR-A and PR-B isoforms), Prlr-C3 (Cat. No. 430798-C3, detects transcript variant 1, long isoform), Oxtr-C4 (Cat. No. 412178-C4), Gal (Cat. No. 400961, 400961-C2) and FlpO (Cat. No. 520791-C3). NeuN antibody (Millipore MAB377) was used at 1:500.

Imaging and image analysis

Images were acquired on a Vectra Polaris Automated Quantitative Pathology Imaging System (Akoya Biosciences) at 20× magnification. Regions of interest (ROIs) were selected using Phenochart software (Akoya Biosciences) and image tiles were spectrally unmixed using inForm Tissue Analysis software (Akoya Biosciences). Stitching of spectrally unmixed image tiles, as well as neuron and subcellular spot detection were performed using custom scripts in QuPath software (*68*). The NeuN channel was used for detection of neuronal cell bodies, and only detections that were also positive for a nuclear marker (DAPI) were retained. Subcellular detection of receptor transcripts was then performed on these neuron

detections. Since every third 18 µm section was processed, the effective spacing between sections was 54 µm. We next used the Fiji plugin ABBA (https://biop.github.io/ijp-imagetoatlas/) to register coronal brain sections to the Allen Brain Atlas (CCFv3) (*69*). Briefly, X and Y rotation were adjusted across all sections from a given brain and two rounds of affine registration using Elastix were performed. Samples then underwent non-rigid registration using the BigWarp tool (sample channel: DAPI, atlas channel: Nissl). Transformed cell detections were exported from QuPath, visualized using a custom Python app (https://github.com/nickdelgrosso/ABBA-QuPath-RegistrationAnalysis), and analyzed using custom Python scripts. To determine a threshold (number of transcripts) for classifying neurons as Esr1-positive, we performed immunostainings against Esr1 in 18 µm vibratome sections throughout the MPOA. A neuronal counterstain (NeuroTrace, see 'Histology and immunostaining') was used to determine the percentage of Esr1-positive neurons in two subregions, MPO and MPN, which were present in all sections, to 40.4 ± 5.9% (means ± SEM, n = 3 mice; fig. S2D). This percentage corresponded to a threshold of ≥ 3 transcripts per cell in our RNAscope dataset. Due to unavailability of commercial PR antibodies that reliably work in brain tissue, we used previously published numbers of PR+ neurons for two hypothalamic areas (ARH and AVPV) as a reference (*70*). Based on these data, a threshold of ≥ 5 transcripts per cell was chosen. The same threshold was used for Prlr and Oxtr. To determine the efficiency of viral-genetic receptor KO, *Esr1*^{loxP} or *PR*^{loxP} animals received unilateral MPOA injections of either AAV2/5-CMV-EGFP-Cre (KO) or AAV2/5-CMV-EGFP (ctrl, fig. S3). Two weeks after viral injection, the numbers of receptor-expressing neurons on the injected and non-injected sides were detected via immunostaining or ISH. Numbers of receptor-expressing neurons (IHC) or average staining intensity values (ISH) were obtained for the injected and non-injected hemisphere in the MPOA using QuPath. Since receptor density varies along the rostrocaudal axis, the ratio of detected cells (or intensity values) between the injected vs non-injected sides was calculated for each section.

Slice physiology

Whole-cell patch clamp recordings

The progeny of Gal-Cre mice crossed to a floxed tdTomato line (Ai9) was used for targeted recordings from $MPOA^{Gal}$ neurons. For recordings of Gal-negative MPOA neurons, tdTomato-negative MPOA neurons were targeted in these mice. Procedures for obtaining MPOA-specific receptor KO or control animals are described in 'Receptor knockout experiments'. Mice were deeply anaesthetized with 3% isoflurane in oxygen and decapitated. The brain was quickly dissected and placed in ice-cold slicing solution containing (in mM): sucrose (213), KCl (2.5), NaH_2PO_4 (1.3), $NaHCO_3$ (26), $MgSO_4$ (2), $CaCl_2$ (2), and D-glucose (10), equilibrated with carbogen (95% O_2 / 5% CO_2). Coronal brain slices (250 µm thickness) containing the MPOA were cut on a vibratome (Leica VT1200S) in ice-cold slicing solution and transferred to an incubation chamber with artificial cerebrospinal fluid (aCSF) containing (in mM): NaCl (127), KCl (2), NaH_2PO_4 (1.2), $NaHCO_3$ (26), $MgSO_4$ (1.3), $CaCl_2$ (2.4) and D-glucose (10), which was continuously oxygenated with carbogen. After at least 1 h of recovery at 37 °C, slices were transferred to a submersion chamber under an upright microscope with infrared Nomarski differential interference contrast optics (Slicescope, Scientifica). During experiments, slices were submerged and continuously perfused (2–3 ml / min) with aCSF at near physiological temperature (33°C) and continuously bubbled with carbogen. Glass micropipettes (3–6 MΩ resistance) were pulled from borosilicate capillaries (World Precision Instruments, Aston, UK) on a P-97 Flaming/Brown micropipette puller (Sutter, Novato, CA) and filled with internal solution containing (in mM): K-Gluconate (140), KCl (10), KOH (1), EGTA (1), Na_2ATP (2), HEPES (10), pH 7.3, 280–290 mOsm. For recording spontaneous excitatory postsynaptic currents (sEPSCs) and inhibitory currents (sIPSCs), voltage clamp recordings were performed at the reversal potential of chloride (−70 mV) or the reversal potential for fast excitatory neurotransmission (0 mV), respectively, using an internal solution containing (in mM): CsMeS (140), CsCl (10), HEPES (10), 120 EGTA (10), and Na_2ATP (2) (pH 7.3 adjusted with KOH). For post hoc reconstruction of neuronal morphology, Neurobiotin (SP-1120, Vector Laboratories) was added at a concentration of 0.2% (w/v). Access resistance was monitored throughout the experiment, and neurons in which the series resistance exceeded 25 MΩ or changed by ≥20% were excluded. Liquid junction potential was 16.4 mV and not compensated. We characterized the intrinsic electrophysiological properties of cells using a standardized current-clamp protocol that consisted of I/V curves, ramps, and current injections. Resting membrane potential was assessed immediately after establishing a whole-cell configuration. To assess excitability, neurons were clamped at -60 mV and ramping depolarizing currents

(10 pA / s) from +25 to +165 pA were injected. Synaptic events (sPSCs) were detected using a threshold-based detector (template mode, WinEDR v4). Rise time is defined as the time needed for sPSC amplitude to reach $1\text{-}e^{-1}$ (≈ 63%) of its maximal value and time constant of decay defined as the time needed for sPSC amplitude to return to $1/e$ (≈ 37%) of resting state. To block GIRK channels, slices were incubated with 100 nM Tertiapin-Q (Tocris 1316) for 1 h before recordings started. Recordings were performed using an Axon Instruments Multiclamp 700B amplifier (Molecular Devices), low pass filtered at 10 kHz, and digitized using a Digidata 1322A digitizer (Molecular Devices). Slow and fast capacitive components were semi-automatically compensated. Offline data analysis was performed with Clampfit 10 software (Molecular Devices), WinEDR v4, WinWCP v5 (http://spider.science.strath.ac.uk/sipbs/software_ses.htm), and custom routines written in Python 3.7.

Morphological reconstructions
After recordings, slices were post-fixed with 4% PFA in PBS with sucrose (200 mM) and HEPES (0.1 M) at 4°C on a nutator o/n, rinsed in PBS and washed in PBS-T for 1 h. Incubation with Streptavidin-647 (Thermo Fisher S32357, 1:2,000) was performed for 24 h at 4°C on a nutator. After washing in PBS-T for 1 h, slices were rinsed in PBS and mounted on slides in DAPI-containing Vectashield mounting medium (Vector Laboratories, H-1200). Brain slices were imaged on a Zeiss LSM 710 confocal microscope, using a 63× (NA = 1.4) oil immersion objective and a *Z* step size of 0.5 µm. Morphological reconstruction of the soma and neurites of each Neurobiotin-filled neuron was performed using Neurolucida software (MBF Bioscience), and spines were reconstructed using the Filament Tracer module (auto-path method, spine diameter 0.25 µm) in Imaris 9.0 (Bitplane). Soma volumes were obtained from non-recorded $MPOA^{Gal}$ neurons in the same image volumes using the Surfaces module in Imaris.

Ex vivo calcium imaging
Brain slices from virgin or D18 Gal-Cre, Ai95 mice were prepared as described above (‘Whole-cell patch clamp recordings’). Spontaneous calcium activity was monitored using a 40× (NA = 0.8) water immersion objective and a Prime 95B sCMOS camera (Teledyne Photometrics) mounted on an Olympus BX51WI microscope (Slicescope, Scientifica). Recordings lasted 5 min. All calcium imaging data were processed using Inscopix Data Processing Software (IDPS, Inscopix Inc.). Data were cropped to areas

containing GCaMP fluorescence. Spatial band-pass Gaussian filtering was used to subtract background. Motion was corrected for each video using the mean image of the entire video as a reference and ΔF/F calculated. Constrained Nonnegative Matrix Factorization (CNMF-E) was used to extract GCaMP6s fluorescence transients from individual neurons. Data were manually inspected for signal from non-somatic compartments or overlapping cells, and traces exported for analysis with custom Python routines.

<u>Miniature microscope imaging</u>

Mice were connected to a miniature microscope (nVista, Inscopix) to check for sufficient expression of GCaMP7s. Imaging data were acquired using nVista HD software (Inscopix) at a frame rate of 20 Hz with 475 nm LED power of 0.1–0.2 mW / mm^2, analog gain of 5–8, and a resolution of 800 × 1,280 pixels. Imaging parameters and focal depth were kept identical across sessions. Imaging and behavioral video collection was synchronized using Bonsai software (Neurogears). Mice were connected to the microscope and allowed 20 min of habituation before recordings were performed in the home cage. A 1 min baseline was acquired before introducing pups, which was used to calculate the relative fluorescence change for each ROI in the field of view (see below).

Preprocessing

Imaging frames were spatially downsampled to 400 × 540 pixels. Drift of the baseline signal over time was removed using a spatial bandpass filter with lower cut-off spatial frequency of 0.005 oscillations per pixel and upper cut-off frequency of 0.5 oscillations per pixel. Motion artifact correction was performed and the relative fluorescence change ΔF for each pixel compared to baseline was calculated as $\frac{\Delta F}{F0} = \frac{F - F0}{F0}$ (where F0 is the mean fluorescence value of each pixel during the baseline period). PCA/ICA-based cell detection was performed using a mean ROI radius of 7–9 pixels in Inscopix Data Processing Software. All automatically identified cells were manually verified to exclude false-positive detections, and cells not detected by the algorithm were manually added. For each verified cell, the relative change in calcium signal $\frac{\Delta F}{F0}$ was then normalized as follows:

$$\frac{\Delta F}{F0} = \frac{\frac{\Delta F}{F0} - \min\left(\frac{\Delta F}{F0}\right)}{\max\left(\frac{\Delta F}{F0}\right) - \min\left(\frac{\Delta F}{F0}\right)}.$$

Evoked activity analysis

Z scores were calculated based on the period of -2 to 0 s before event detection (baseline period) and 0 to 2 s from event detection (activity period) as $z = \frac{x - \mu}{\sigma}$, where x is ΔF/F of the current timestamp, μ is the mean ΔF/F of the baseline period and σ is the standard deviation of the baseline period. Significant responses were called when the *z*-scored ΔF/F of the baseline and activity periods were significantly different (unpaired *t* test). ROIs were thereafter categorized as exhibiting increased, decreased, or unchanged evoked activity. The single neuron tuning index was derived from performing an unpaired *t* test between the activity and baseline periods. Tuning indices were normalized based on their absolute value using a min max scaler from the sklearn preprocessing package while keeping their original sign.

Selectivity index

In order to assess selectivity, stimuli were presented sequentially and in randomized order. To obtain the selectivity of individual neurons for pups vs other social and non-social stimuli, the choice probability for each neuron was calculated (*71*). This procedure uses ΔF/F values of single neurons during pairs of chemoinvestigation behaviors (e.g., investigation of pups vs intruders) to estimate how likely two behaviors can be distinguished given their respective ΔF/F distributions. The first sniffing episode towards each stimulus was used here to obtain better separation between activity transients evoked by different stimuli in sniffing sequences, and since we observed a gradual desensitization during repeated sniffing of the same stimulus (fig. S11F). To calculate the selectivity index, ΔF/F distributions were first plotted against each other as histogram pairs. Histograms were then plotted against each other to generate a ROC curve, and the selectivity index calculated as: $1 - (area\ under\ the\ ROC\ curve)$. Neurons exclusively active during pup sniffing would thus have a selectivity index of 1, neurons exclusively active during sniffing of another stimulus a selectivity index of 0, and non-selective neurons a selectivity index of 0.5.

Linear discriminant analysis

To assess the separability of social and non-social stimuli by the MPOA^{Gal} population, we performed linear discriminant analysis (LDA), using timestamps as features (*72*). LDA is a supervised dimensionality reduction method which projects input data into a subspace, the axes of which maximize separation between classes. LDA was computed on each individual recording using two inputs: (1) a 3D array x: the population dynamics of all n neurons from a recording 3 s after the onset of k behaviors (first pup sniff, female and male intruder sniff, screw sniff, dummy pup sniff and food pellet sniff), forming $k = 4$ classes. Results were similar when the second sniffing episode for each stimulus was used (fig. S11, G and H). With a sampling of 20 Hz over 3 s, this results in a matrix with a dimension of $n \times k \times 60$. (2) Vector y: the second input assigns behavior labels to each of k activity episodes in x, giving a k-dimensional vector. Using these two pieces of information, LDA maximizes the posterior log likelihood of input y belonging to class i out of k classes computed as $\log P\,(y = i|x) = \omega_i^k x + \omega_{i0} + Cst$ where $\omega_i = \Sigma^{-1}\mu_i$ and $\omega_{i0} = -\frac{1}{2}\mu_i^k\ \Sigma^{-1}\mu_i + \log P\,(y = i)$. This can be achieved by minimizing the term $(x - \mu_i)^k\ \Sigma^{-1}(x - \mu_i)$ which will minimize both the variance within each class and the distances between x and each of the k classes. The sklearn linear discriminant classifier was used for this analysis with an 'eigen' solver and a 0.001 shrinkage parameter for regularization. For better visualization, ellipses comprising the 95% confidence intervals were fitted to LDA clusters. To quantify separability of LDA clusters, all episodes in x were again fitted to the derived LDA space with their class assigned by the fitted LDA model. The Rand index (RI) was calculated based on this predicted class and ground truth class y as follows: $RI = \frac{No.agreements\,(TP+TN)}{No.agreements\,(TP+TN) + No.disagreements\,(FP+FN)}$ where TP = true positive, TN = true negative, FP = false positive, and FN = false negative.

Quantification and statistical analyses

For details of statistical testing see figure legends. All test statistics and *P* values were calculated using the SciPy stats module (1.7.3) . All data are expressed as mean ± SEM unless stated otherwise. Box plots represent the median and 25th and 75th percentiles, and whiskers represent the data range. Two-sided Mann Whitney U test was used to compare two independent groups. Wilcoxon signed-rank test or paired *t* test was used for paired data based on distribution and sample size. When >2 groups were compared, ANOVA was performed, followed by the Tukey HSD *post hoc* test. When several groups were compared

to a single reference group (e.g., Vir), Dunnett's *post hoc* test was used instead. For longitudinal analysis of behavioral data, repeated-measures ANOVA was used. Two-tailed Fisher's exact test was used for categorical data, and Benjamini-Hochberg false discovery rate adjustment was performed for multiple comparisons (FDR < 0.05). Kaplan-Meier (K-M) survival analysis was performed to evaluate behavioral latencies which contained saturated data. The K-M estimator was computed using occurrence of retrieval (or contact) as event type 1; retrieval (or contact) failure over the course of the assay constituted a right censored event (event type 0). Pairwise *P* values were obtained by log-rank test on K-M curves and adjusted for multiple comparisons using the Benjamini-Hochberg FDR procedure. For comparing two distributions, the Kolmogorov–Smirnov test was used. Throughout the study, $P < 0.05$ was considered statistically significant. In initial experiments, no statistical methods were used to pre-determine sample sizes, but sample sizes were similar to those reported in similar studies. In follow-up experiments, minimum sample sizes were predetermined from power estimates based on these initial experiments. Experiments were randomized and the investigators were blinded to allocation during experiments and outcome assessment, except where this was not possible, e.g., when scoring behaviors of visibly pregnant females.

Supplementary Text

Brain areas

Brain area annotations are from the Allen Mouse Brain Common Coordinate Framework (CCFv3) (*6*). Abbreviations as follows: ADP, Anterodorsal preoptic nucleus, AHN, Anterior hypothalamic nucleus, ARH, Arcuate hypothalamic nucleus, AVP, Anteroventral preoptic nucleus, AVPV, Anteroventral periventricular nucleus, DMH, Dorsomedial nucleus of the hypothalamus, HY, Hypothalamus, LPO, Lateral preoptic area, MEPO, Median preoptic nucleus, MMme, Medial mammillary nucleus, median part, MPN, Medial preoptic nucleus, MPO, Medial preoptic area, PD, Posterodorsal preoptic nucleus, PeF, Perifornical nucleus, PH, Posterior hypothalamic nucleus, PMd, Dorsal premammillary nucleus, PMv, Ventral premammillary nucleus, PS, Parastrial nucleus, PVa, Periventricular hypothalamic nucleus - anterior part, PVi, Periventricular hypothalamic nucleus - intermediate part, PVp, Periventricular hypothalamic nucleus - posterior part, PVpo, Periventricular hypothalamic nucleus - preoptic part, PVH, Paraventricular hypothalamic nucleus, RCH, Retrochiasmatic area, SBPV, Subparaventricular zone, SUM, Supramammillary nucleus, TMd, Tuberomammillary nucleus - dorsal part, TMv, Tuberomammillary nucleus - ventral part , TU, Tuberal nucleus, VMH, Ventromedial hypothalamic nucleus.

Number of $MPOA^{Gal}$ neurons

We observed an increase in the number of MPOA neurons expressing Galanin mRNA in pregnant females (Vir: 127 ± 11 neurons / mm^2; D18: 174 ± 14 neurons / mm^2; 24 and 29 sections from n = 4, 5 mice; $P = 0.023$, U test). We also quantified numbers of tdTomato+ neurons in the MPOA of Gal-Cre, tdTomato mice which were used for targeted whole-cell patch clamp recordings (Fig. 2A, *upper panel*) and found no significant difference between virgins and pregnant females (Vir: 150 ± 25 neurons / mm^2; D18: 178 ± 24 neurons / mm^2; 16 sections from n = 4 mice each; $P = 0.32$, U test). For all other electrophysiological and *in vivo* imaging experiments, AAVs expressing Cre-recombinase and/or EGFP, or GCaMP were injected 2 weeks (electrophysiology) or 6–8 weeks (imaging) prior to animals becoming pregnant. Any onset of Galanin expression in additional MPOA neurons during pregnancy does therefore not affect these experiments.

Resting membrane potential (RMP) of MPOA^{Gal} neurons

MPOA^{Gal} neurons have comparatively high RMP (Vm, Vir: −42.9 ± 0.9 mV, D18: −46.7 ± 1.1 mV, Fig. 2C). To our knowledge, this is the first electrophysiological characterization of this cell type in females. We therefore addressed whether these values might be high due to our experimental conditions and recorded from primary motor cortex (M1) layer 5 pyramidal cells in the same coronal slices that we prepared for MPOA recordings (fig. S6X). All solutions, experimental procedures and recording parameters were otherwise identical. These recordings yielded an average RMP of −69.7 ± 3.4 mV (fig. S6X) which closely aligns with previously reported values for layer 5 M1 neurons (e.g. see (*73*). We therefore conclude that a high RMP is a genuine property of MPOA^{Gal} neurons in females.

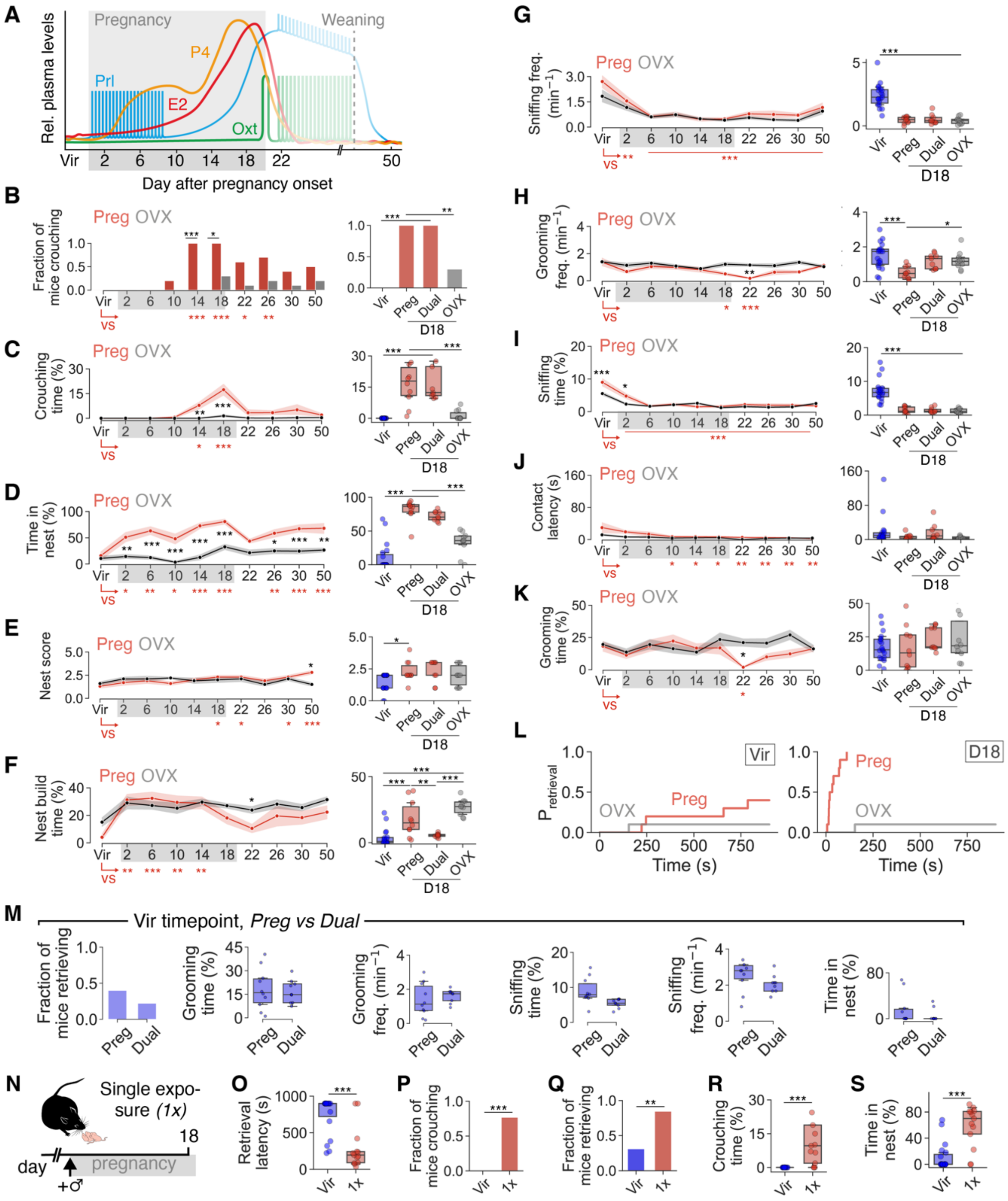
A
Rel. plasma levels
Pregnancy
Weaning
P4
Prl
E2
Oxt
Vir 2 6 10 14 18 22 50
Day after pregnancy onset
B
Fraction of mice crouching
Preg OVX
vs
D18
Vir Preg Dual OVX
C
Crouching time (%)
D
Time in nest (%)
E
Nest score
F
Nest build time (%)
G
Sniffing freq. (min−1)
H
Grooming freq. (min−1)
I
Sniffing time (%)
J
Contact latency (s)
K
Grooming time (%)
L
Pretrieval
Vir
D18
Time (s)
M
Vir timepoint, Preg vs Dual
Fraction of mice retrieving
Grooming time (%)
Grooming freq. (min−1)
Sniffing time (%)
Sniffing freq. (min−1)
Time in nest (%)
N
Single expo-sure (1x)
day
18
pregnancy
+♂
O
Retrieval latency (s)
P
Fraction of mice crouching
Q
Fraction of mice retrieving
R
Crouching time (%)
S
Time in nest (%)
Vir 1x

Fig. S1. Hormonal changes during pregnancy, and pregnancy-associated changes in pup-directed behavior.

(**A**) Relative plasma levels of canonical pregnancy hormones in mice. Data obtained and redrawn from refs. (*74, 75*). (**B** to **K**) Parental behavior parameters that are primarily affected by pregnancy-associated hormonal changes (B to E), primarily affected by frequent pup exposure (F to J), or unaffected (K). Within-group (*Preg*, virgin [Vir] vs subsequent timepoints, red asterisks) and between-group (*Preg* vs *OVX*, black asterisks) comparisons shown. (**L**) Kaplan-Meier survival curves for retrieval probabilities at Vir and D18 in *Preg* (n = 10) and *OVX* (n = 10) groups. (**M**) Comparison of pup-directed behaviors of virgins in *Preg* (n = 10) and *Dual* (n = 9) groups. Fisher's exact test (fraction retrieving) or U test. (**N**) Pup-directed behaviors in females exposed to pups only at D18 (*1x*). (**O** to **S**) Behaviors of virgins (pooled from *Preg* and *Dual* groups, n = 19) and *1x* females at D18 (n = 13). Repeated-measures ANOVA with Dunnett's *post hoc* test for within-group comparisons, and one-way ANOVA with Tukey *post hoc* test for between-group comparisons in (B) to (I) and (K). Kaplan-Meier survival analysis with log rank test in (J) and (O), Fisher's exact test in (B), (P) and (Q) (right), U test in (R) and (S). Grey boxes in (A) to (K) highlight pregnancy. Shaded areas and error bars in (B) to (K) SEM, solid lines represent mean values. ***$P < 0.001$, **$P < 0.01$, *$P < 0.05$.

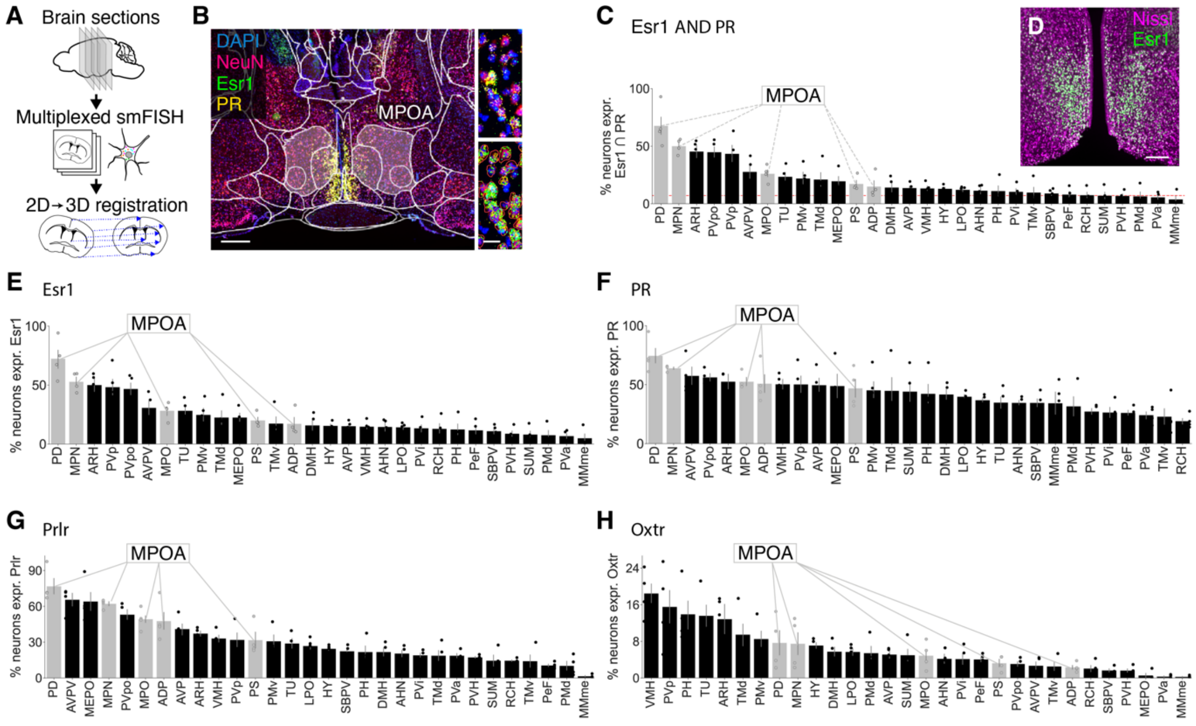

Fig. S2. Pregnancy hormone receptor expression in hypothalamic areas.

(**A**) Multiplexed smFISH detection of hormone receptor transcripts in brain sections, and registration to brain atlas. (**B**) Example coronal brain section with Esr1 and PR transcripts, and DAPI and NeuN counterstains. Insert shows example neuronal and subcellular detections. (**C**) Percentage of neurons co-expressing Esr1 and PR mRNA in hypothalamic areas. MPOA subregions highlighted (n = 4 mice, 2 virgins and 2 D18 females). Red line, brain-wide average. (**D**) Esr1 immunostaining and NeuN counterstain in MPOA. (**E** to **H**) Percentage of neurons expressing Esr1 (E), PR (F), prolactin receptor (Prlr, G) or oxytocin receptor (Oxtr, H) mRNA in hypothalamic areas (n = 4 mice, 2 virgins and 2 D18 females). MPOA subregions in gray: PD, posterodorsal preoptic nucleus, PS, parastrial nucleus, MPN, medial preoptic nucleus, ADP, anterodorsal preoptic nucleus, MPO, medial preoptic area. For other brain area abbreviations see Supplementary Text. Error bars are SEM. Scale bars, 500 µm (B), 20 µm (B, inserts) and 200 µm (D).

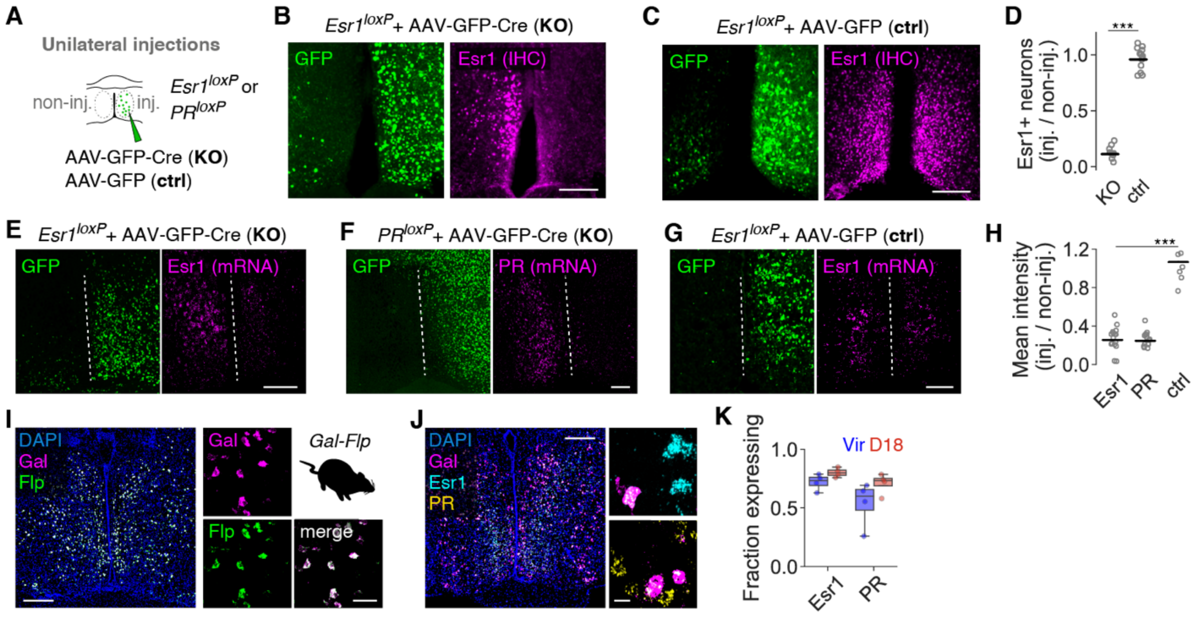

Fig. S3. Validation of viral-genetic receptor ablation, validation of Gal-Flp mice, and receptor expression in MPOA^{Gal} neurons.

(**A**) Unilateral viral-genetic ablation (KO) of Esr1 or PR from MPOA neurons, and negative control (ctrl). (**B** and **C**) Example MPOA sections with Esr1 immunostaining in injected and non-injected hemisphere of KO (B) and ctrl (C) animals. (**D**) Ratio of Esr1 immunopositive neurons in injected vs non-infected hemisphere (14 sections, n = 3 mice each, $P = 4.71 \times 10^{-5}$, U test). (**E** to **G**) Example MPOA sections of unilateral Esr1 KO (E), PR KO (F), and ctrl (G) with smFISH against receptor transcripts and GFP. (**H**) Ratio of relative staining intensity in injected vs non-infected hemisphere (15 sections, n = 2 mice each). (**I**) Verification of Flp recombinase expression in MPOA^{Gal} neurons by smFISH against Gal and Flp transcripts (specificity: 99.3% of Flp+ cells Gal+, 2,967 cells; efficiency: 98.5% of Gal+ cells Flp+, 2,531 cells; n = 2 mice). (**J**) Example coronal brain section with Esr1 and PR mRNA expression in MPOA^{Gal} neurons. (**K**) Percentage of MPOA^{Gal} neurons expressing Esr1 or PR transcripts in virgins and D18 females (n = 4 mice each, $P = 0.11$ and 0.11, U test). Data in (A) to (H) are from D18 females. Scale bars, 200 µm in (B), (C) and (E) to (G), 250 µm in (I) and (J), 50 µm in (I, inserts), 20 µm in (J, inserts).

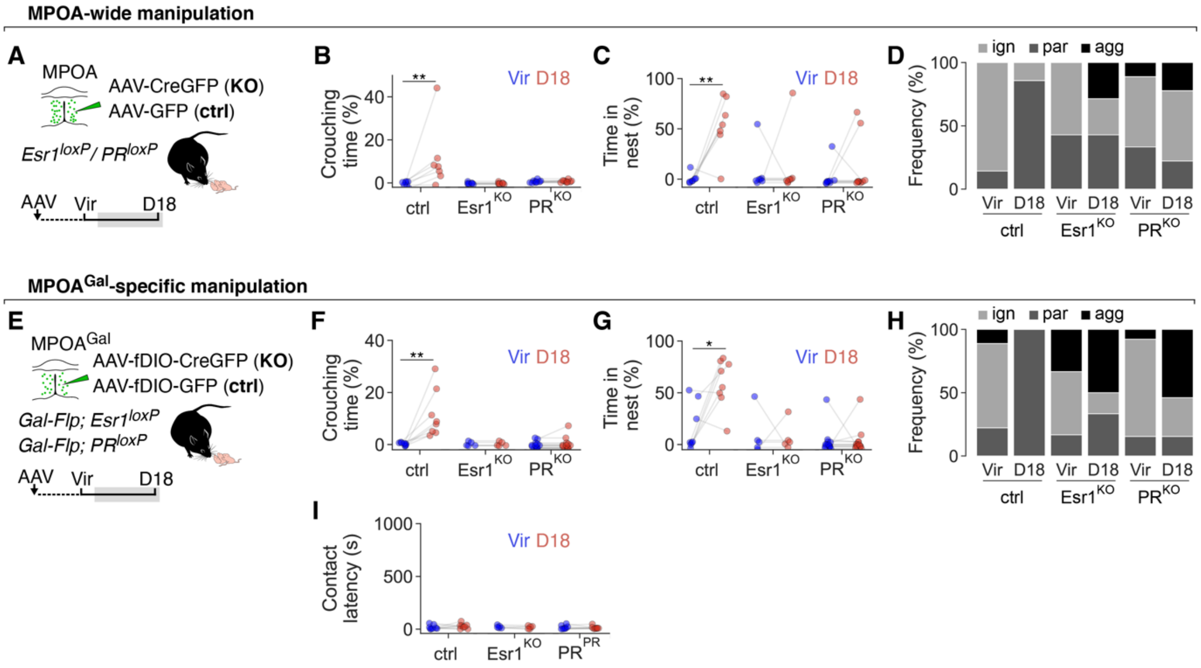

Fig. S4. Behavioral effects of ablating hormone receptors in MPOA$^{(Gal)}$ neurons.

(**A** and **D**) Targeted ablation (KO) of Esr1 or PR from all MPOA neurons (A) or from MPOA^{Gal} neurons (D) and negative controls (ctrl). (**B** to **D**, **F** to **I**) Effects of ablating Esr1 or PR across MPOA (B to D, n = 7, 8, 9 mice) or selectively in MPOA^{Gal} neurons (F to I, n = 8, 5, 13 mice) on pup-directed behaviors. In (D) and (H), ign, ignoring; par, parental; agg, aggressive. One-way ANOVA with Tukey HSD *post hoc* test in (B), (C), (F), (G) and (I). Fisher's exact test with Benjamini-Hochberg FDR correction in (D) and (H). $^{***}P < 0.001$, $^{**}P < 0.01$, $^{*}P < 0.05$.

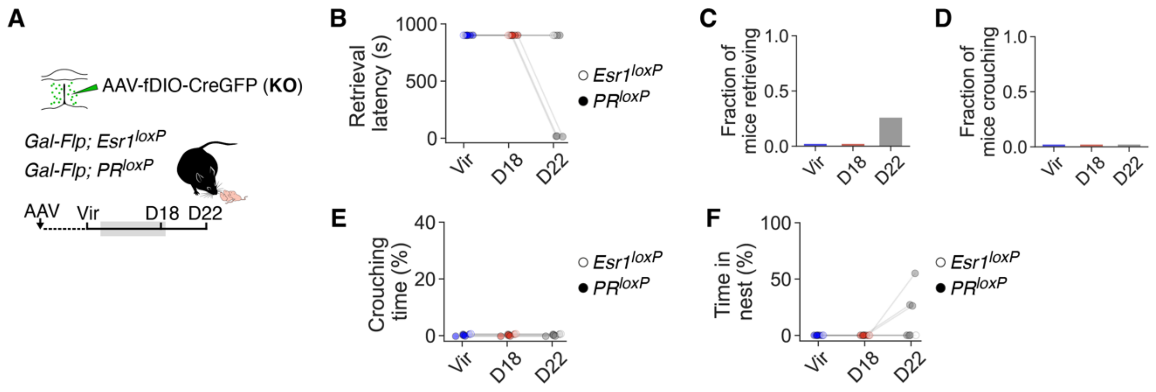

Fig. S5. Behavioral effects of hormone receptor KO in MPOA^{Gal} neurons persist in the postpartum period.

(**A**) Targeted ablation (KO) of Esr1 or PR from MPOA^{Gal} neurons. (**B** to **F**) Effects of ablating Esr1 or PR in MPOA^{Gal} neurons on pup-directed behaviors at D18 and in early postpartum (D22, n = 7 [1 *Esr1^{loxP}* and 6 *PR^{loxP}* mice]). Empty circles in (B), (E) and (F), *Esr1^{loxP}* mice, filled circles *PR^{loxP}* mice. Kaplan-Meier survival analysis with log rank test in (B), Fisher's exact test with Benjamini-Hochberg adjustment for multiple comparisons in (C) and (D), one-way ANOVA in (E) and (F). ***P < 0.001, **P < 0.01, *P < 0.05.

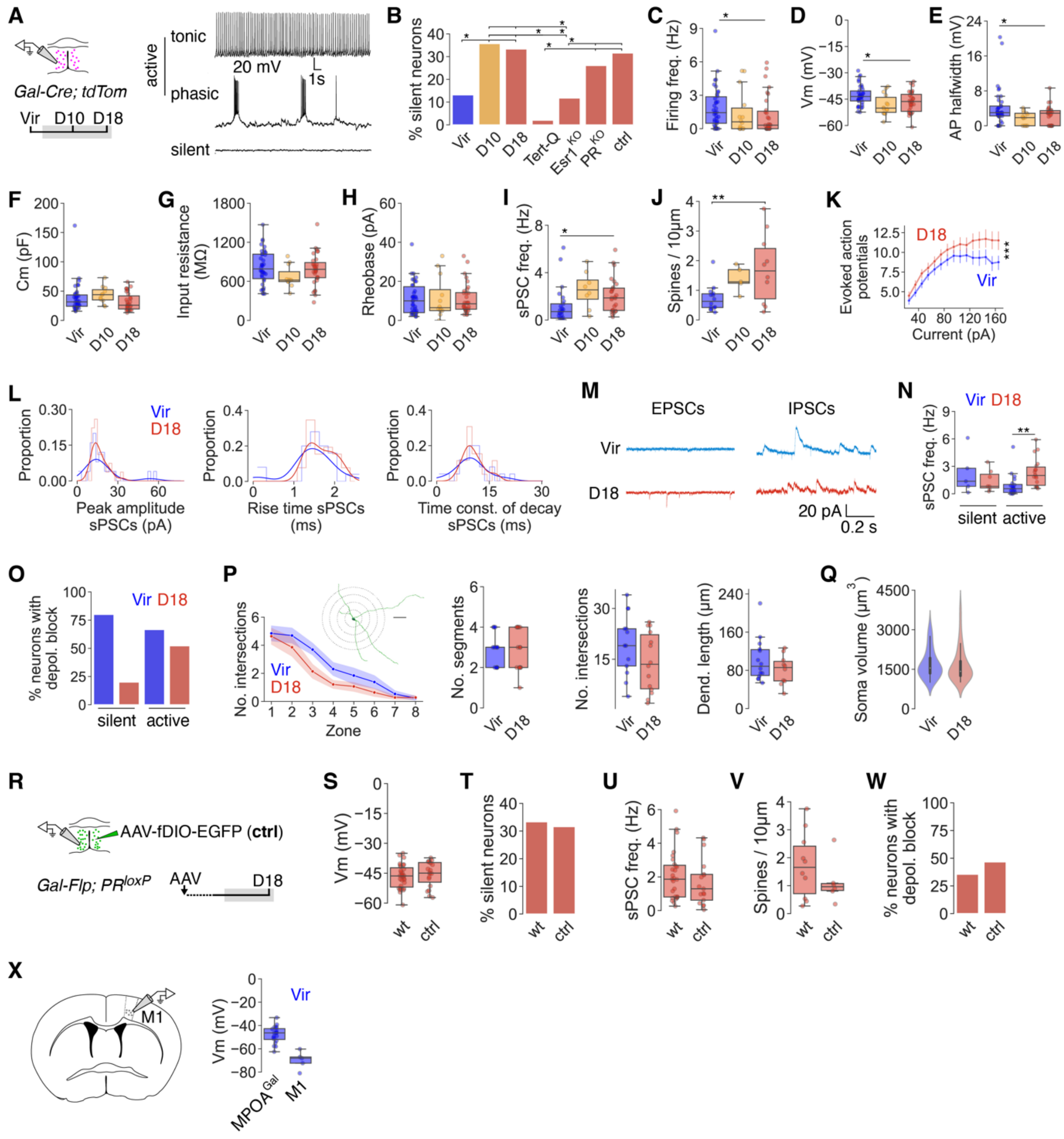

Fig. S6. Pregnancy-induced biophysical and morphological changes in MPOA^{Gal} neurons.

(**A**) Whole-cell recordings in brain slices from wild-type MPOA^{Gal} neurons and representative example traces of different activity patterns of MPOA^{Gal} neurons at resting membrane potential. (**B**) Percentage of

silent neurons at resting membrane potential (38, 14, 30, 9, 17, 23, 19 cells from n = 15, 4, 9, 3, 3, 5, 4 mice). Tert-Q, Tertiapin-Q. (**C** to **H**) Spontaneous firing frequency (C), resting membrane potential (D), action potential half-width (E), membrane capacitance (F), input resistance (G) and rheobase at -60 mV (H) across groups (38, 14, 31 cells from n = 15, 4, 8 mice). (**I** and **J**) sPSC frequency (I, 21, 8, 23 cells from n = 9, 6, 6 mice) and spine density (J, 14, 5, 10 cells from n = 10, 4, 4 mice). (**K**) Evoked action potentials per injected somatic current. (**L**) sPSC amplitude, rise time and decay time (21, 23 cells from n = 9, 6 mice). (**M**) Example voltage clamp traces from $MPOA^{Gal}$ neurons with EPSCs and IPSCs. (**N**) sPSC frequency in silent vs active cells (5, 10, 33, 20 cells). (**O**) Percentage of silent vs active neurons exhibiting depolarization block (5, 10, 33, 23 cells). (**P**) Measures for dendritic complexity of reconstructed $MPOA^{Gal}$ neurons. Number of intersections with circular 50 µm zones around the soma (Sholl analysis; example neuron), number of unique segments, total number of intersections, and dendritic length for neurons from virgins and D18 females (13, 14 neurons from n = 8, 5 mice). (**Q**) $MPOA^{Gal}$ soma volume in Vir and D18 females (55, 58 neurons from n = 3 mice each). (**R**) Whole-cell recordings in brain slices from $MPOA^{Gal}$ neurons transduced with control AAV. (**S** to **W**) D18 recordings of wild-type (wt) vs control AAV (ctrl) $MPOA^{Gal}$ neurons. Resting membrane potential (S, 38, 19 cells from n = 15, 4 mice), percentage of silent neurons at resting membrane potential (T, 38, 19 cells from n = 15, 5 mice), sPSC frequency (U, 21, 19 cells from n = 9, 4 mice), spine density (V, 14, 6 cells from n = 10, 2 mice) and percentage of neurons exhibiting depolarization block (W, 34, 30 cells from n = 15, 8). (**X**) Control recordings from layer 5 pyramidal cells in primary motor cortex of virgin females (M1, 5 cells from n = 2 mice). Fisher's exact test in (B), (O), (T) and (W), Benjamini-Hochberg adjustment for multiple comparisons in (B) and (O). One-way ANOVA with Dunnett's *post hoc* test in (C) to (J). Two-way ANOVA with Tukey *post hoc* test in (K) and (P). U test in (L), (P), (Q), (S), (U), (V) and (X). Scale bar in (P), 100 µm. $***P < 0.001$, $**P < 0.01$, $*P < 0.05$.

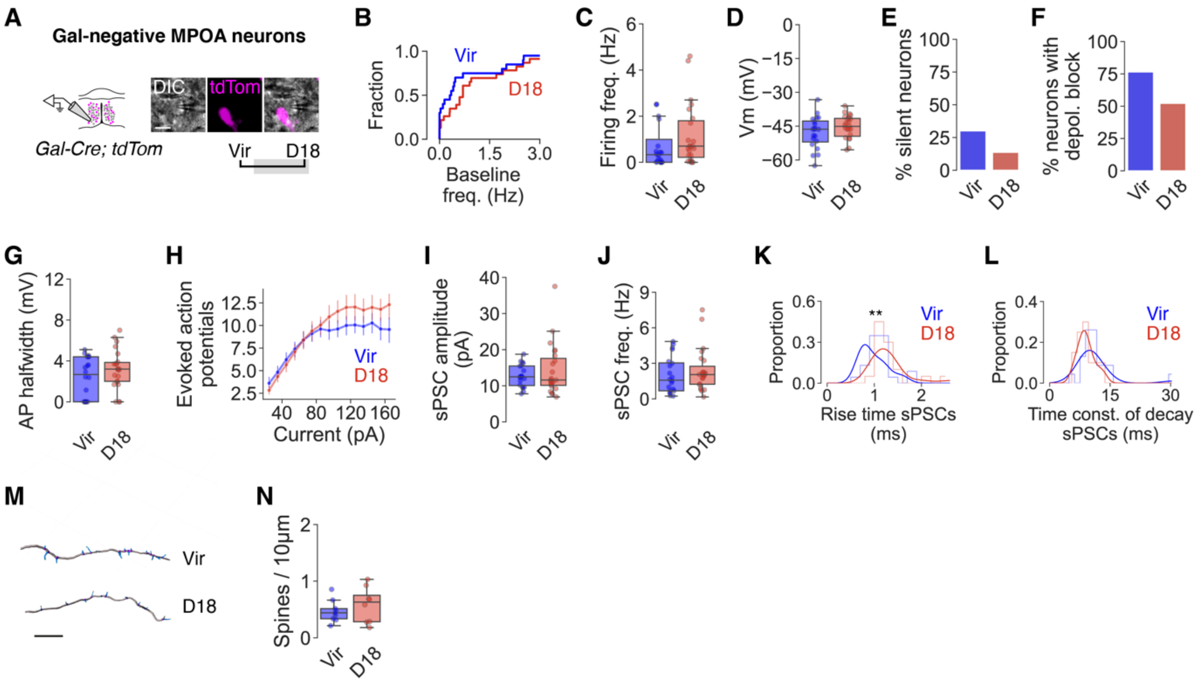

Fig. S7. Absence of equivalent pregnancy-induced changes in Gal-negative MPOA neurons.
(**A**) Whole-cell recordings from Gal-negative MPOA neurons in brain slices. (**B** to **L**) Spontaneous firing frequency (B and C), resting membrane potential (D), percentage of silent neurons at resting membrane potential (E), percentage of neurons exhibiting depolarization block (F), action potential half-width (G), evoked spikes per injected current (H), sPSC amplitude (I), sPSC frequency (J), sPSC rise time (K) and sPSC decay time (L) in virgins and D18 females (21, 23 cells from n = 5, 3 mice). (**M**) Dendritic segments of Gal-negative MPOA neurons with spines. (**N**) Spine density in virgins and D18 females (9, 8 cells from n = 4, 3 mice). U test in (B) to (G), (I) to (L) and (N). Two-way ANOVA in (H). Scale bars, 20 µm (A), 10 µm (M). ****P* < 0.001, ***P* < 0.01, **P* < 0.05.

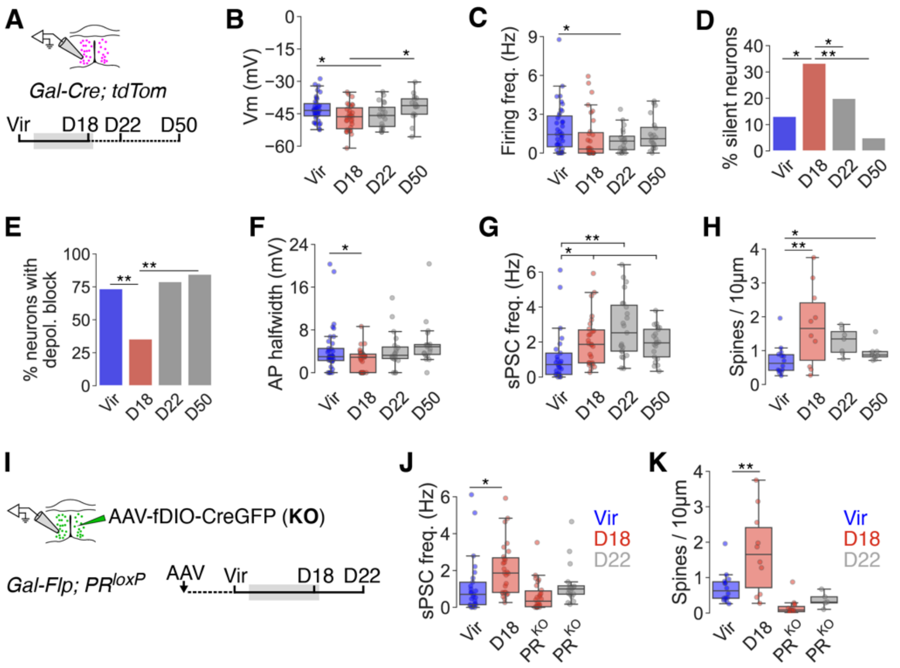

Fig. S8. Off-dynamics of hormone-mediated MPOA^{Gal} neuronal plasticity.

(**A**) Whole-cell recordings in brain slices from wild-type MPOA^{Gal} neurons at D18 and in the postpartum period. (**B** to **H**) Resting membrane potential (B), spontaneous firing frequency (C), percentage of silent neurons at resting membrane potential (D), percentage of neurons exhibiting depolarization block (E), action potential half-width (F) and sPSC frequency (G) across groups (34, 30, 24, 14 cells from n = 15, 8, 5, 5 mice). (**H**) Dendritic spine density (14, 10, 7, 9 cells from n = 10, 4, 4, 4 mice). (**I**) Whole-cell recordings from receptor-deleted (KO) MPOA^{Gal} neurons in early postpartum (D22). (**J** and **K**) sPSC frequency (J) and dendritic spine density (K) of PR-deleted neurons at D22 (34, 30, 15, 5 cells from n = 15, 8, 4, 3 mice). Fisher's exact test with Benjamini-Hochberg adjustment for multiple comparisons in (D) and (E), one-way ANOVA with Dunnett's *post hoc* test in (B), (C), (F) to (H), (J) and (K). ***$P < 0.001$, **$P < 0.01$, *$P < 0.05$.

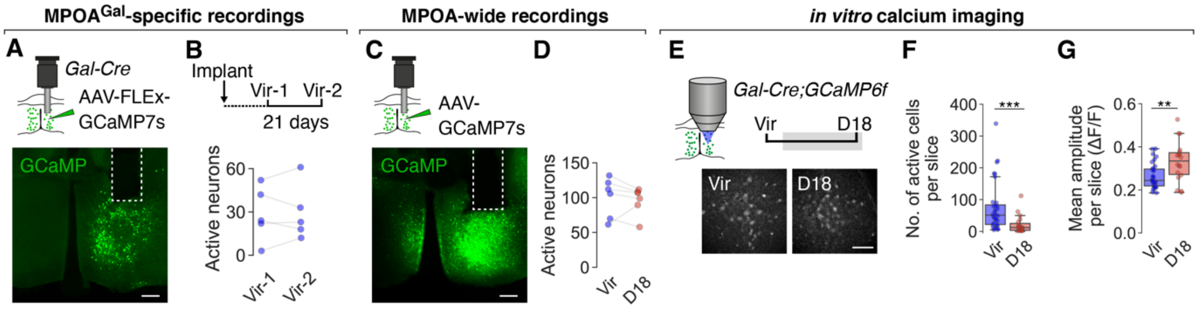

Fig. S9. Controls for MPOA^{Gal} population recordings.

(**A**) Representative GCaMP7s expression and GRIN lens position for recording from MPOA^{Gal} neurons. (**B**) Control group of Gal-Cre animals injected into the MPOA with AAV-FLEx-GCaMP7s, implanted with a GRIN lens, and tested 6 (Vir-1) and 9 (Vir-2) weeks after surgery without experiencing pregnancy. Number of detected neurons per mouse (n = 5 mice). (**C**) Representative GCaMP7s expression and GRIN lens position for pan-MPOA recordings. (**D**) Number of detected neurons per mouse for MPOA-wide recordings (Vir: 100 ± 11 neurons, D18: 95 ± 8 neurons; n = 6 mice). (**E**) Imaging spontaneous MPOA^{Gal} neuronal activity in brain slices. Example frames from recordings in samples from virgins and D18 females are shown. (**F** and **G**) Number of detected cells per slice in virgins and at D18 (F) and average amplitude of calcium transients (G, 2,431 and 414 cells from n = 11, 8 mice). Paired *t* test in (B) and (D). U test in (F) and (G). Scale bars, 250 µm in (A) and (C), 100 µm in (E). ***$P < 0.001$, **$P < 0.01$, *$P < 0.05$.

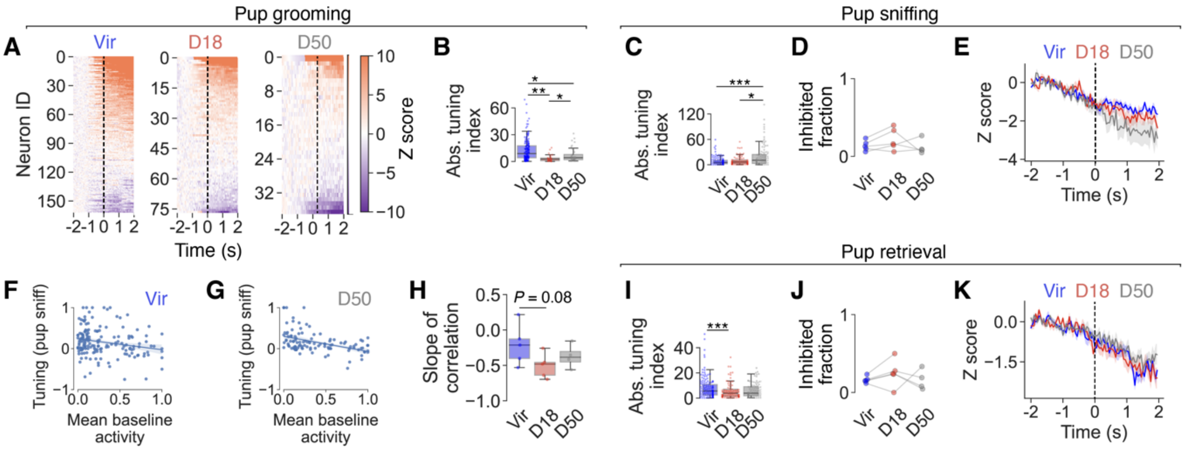

Fig. S10. Additional parameters for MPOA^{Gal} neuronal activity during pup interactions.

(**A**) Temporal profile of responses evoked by pup grooming in virgins and at D18 and D50. Dashed lines indicate action onset. Neuron order based on hierarchical clustering sorted by mean cluster response onset. (**B**) Tuning index for individual neurons to pup grooming. (**C** and **I**) Tuning index for individual neurons to pup sniffing (C) and pup retrieval (I). (**D** and **J**) Fraction of neurons with negative evoked response during pup grooming (D) or retrieval (J, n = 5 mice). (**E** and **K**) Averaged Z score for neurons inhibited in virgins, at D18 and D50 during pup grooming (E, 24, 16, 11 neurons from 5, 5, 4 mice) or retrieval (K, 27, 20, 14 neurons from 5, 5, 4 mice). (**F** and **G**) Correlation between normalized tuning index for responses to pup sniffing and normalized mean baseline activities in virgins ($r^2 = 0.04$, $P < 0.005$) and at D50 ($r^2 = 0.194$, $P < 1.1 \times 10^{-6}$). (**H**) Slope of correlation between tuning index for responses to pup sniffing and normalized baseline activities (see [(F) and (G)]), per mouse). Mixed linear model in (B), (C) and (I). Paired *t* tests in (D) and (J). Linear regression in (F) and (G). Unpaired *t* test in (H). ***$P < 0.001$, **$P < 0.01$, *$P < 0.05$.

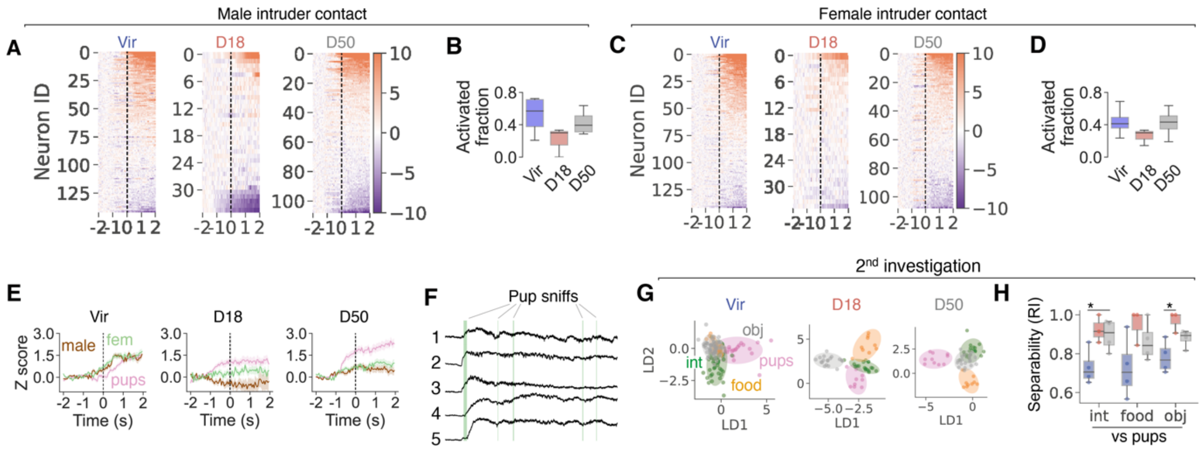

Fig. S11. Additional data for MPOA^{Gal} selectivity and stimulus separability.

(**A** and **C**) Temporal profile of MPOA^{Gal} neuronal responses evoked by contact with male intruder (A) or female intruder (C) in virgins and at D18 and D50. Neuron order based on hierarchical clustering sorted by mean cluster response onset. (**B** and **D**) Fraction of neurons with positive evoked response during sniffing of male (B) or female (D) intruder (n = 5 mice). (**E**) Averaged Z score for MPOA^{Gal} neuronal responses to sniffing pups, male and female intruders in virgins, at D18 and D50. (**F**) Representative activity traces from 5 individual neurons with pup sniffing episodes highlighted. (**G**) Example MPOA^{Gal} neuronal activities at Vir, D18 and D50 during object investigation (sniffing) in LDA space (int, intruder; obj, screw, dummy pup). The second investigation episode for each stimulus was used. Ellipsoids represent 95% confidence area of neuronal activity to each stimulus. (**H**) Separability of indicated stimulus combinations by the MPOA^{Gal} population (RI, Rand Index, n = 4, 3, 4 mice). Dashed lines in (A), (C) and (E) indicate action onset. Paired *t* tests in (B) and (D), unpaired *t* tests in (H). ****P* < 0.001, ***P* < 0.01, **P* < 0.05.

Movie S1.

Ablation (KO) of Esr1 in MPOA neurons abolishes pregnancy-induced onset of parenting. Pup-directed behaviors of a D18 C57BL/6J female (wild-type, wt) and a D18 $\textit{Esr1}^{loxP}$ female with MPOA-wide KO of Esr1 are shown. Note that parts of the video are sped up 8-fold.
